# The prognostic value of the tumor-stroma ratio compared to tumor-infiltrating lymphocytes in triple-negative breast cancer: a review

**DOI:** 10.1007/s00428-025-04039-z

**Published:** 2025-02-04

**Authors:** Layla Andour, Sophie C. Hagenaars, Barbara Gregus, Anna Mária Tőkes, Zsófia Karancsi, Rob A. E. M. Tollenaar, Judith R. Kroep, Janina Kulka, Wilma E. Mesker

**Affiliations:** 1 Department of Surgery, Leiden University Medical Center, Albinusdreef 2, 2333 ZA Leiden, The Netherlands; 2 Department of Pathology, Forensic and Insurance Medicine, Semmelweis University, Budapest, Hungary; 3 Department of Medical Oncology, Leiden University Medical Center, Leiden, The Netherlands

**Keywords:** Breast cancer, Tumor-stroma ratio, Tumor-infiltrating lymphocytes, CD-marker, Prognosis

## Abstract

Previous literature extensively explored biomarkers to personalize treatment for breast cancer patients. The clinical need is especially high in patients with triple-negative breast cancer (TNBC) due to its aggressive nature and limited treatment modalities. This review aims to evaluate the value of tumor-infiltrating lymphocytes (TILs) and tumor-stroma ratio (TSR) as prognostic biomarkers in TNBC patients and assess their clinical potential. A literature search was conducted in PubMed, Embase, Emcare, Web of Science, and Cochrane Library. Papers comparing survival outcomes of TNBC patients with low/high or negative/positive TSR and immune cells were included. The most frequently mentioned subgroups of TILs were selected and reported in this review. Data from 43 articles on TILs and eight articles on TSR were included. Among TNBC patients, high CD8 expression was generally associated with better survival. Notable, the poor survival outcomes were related to high intra-tumoral PD-L1 expression, whereas high stromal PD-L1 expression more often was correlated with favorable outcomes. For the TSR, a high amount of stroma in the primary tumor of TNBC patients was consistently associated with worse survival. This review highlights that a high number of CD8-positive T-cells is a promising prognostic factor for TNBC patients. PD-L1 expression analyzed for intra-tumoral and stromal expression separately reports strong but contrasting information. Finally, the TSR shows potential to be an important prognostic marker, especially for TNBC patients. Utilizing both biomarkers, either on itself or combined, could enhance clinical decision-making and personalization of treatment.

**Supplementary Information:**

The online version contains supplementary material available at 10.1007/s00428-025-04039-z.

## Introduction

Over the years, research on prognostic and predictive markers for breast cancer primarily focused on cancer cells. However, recent insights showed that the tumor microenvironment (TME) is more important for tumor progression than previously fathomed [1]. The TME consists of a wide array of immune cells, fibroblasts, endothelial cells, blood vessels, and the extracellular matrix (ECM) [2]. Within the tumor microenvironment, fibroblasts are ubiquitous. A large part of these fibroblasts obtain a certain modified, activated phenotype, the so-called cancer-associated fibroblasts (CAFs), which largely shape the tumor stroma [3]. CAFs are involved during all stages of tumor development: they are known for playing a significant role in cancer initiation, progression, growth, and spread [4]. By producing tumor-promoting growth factors, such as epidermal growth factor (EGF), transforming growth factor beta (TGF-β), chemokines, and the ECM, CAFs contribute to angiogenesis and metastasis of the tumor cells [5]. It is thought that the cancer cells and cancer stem cells in the TME, along with the associated stroma, play an important role in carcinogenesis [6]. These components hold prognostic and response-predictive information and can, therefore, be used as a potential target in the treatment of cancer. This information can then be used in a clinical setting to select the right treatment for the individual patient.

Current treatment regimens for breast cancer are mainly based on tumor subtypes. However, mutations causing resistance for targeted treatments and intra-tumoral heterogeneity hamper the ability to accurately predict prognosis and the most appropriate treatment [7]. Standard therapy might result in under- or overtreatment in individual cases [8]. Therefore, optimization of personalized treatment is highly valuable in breast cancer patients [7, 9]. This clinical need is especially high in the triple-negative breast cancer (TNBC) subtype, due to its worse prognosis and limited therapy options [10, 11]. One example of personalized therapy is the rapidly emerging treatment modality immunotherapy. Research over the years extended knowledge of the interaction between tumor cells and the microenvironment of the tumor, especially cells related to the immune system. Immunotherapy is a possible therapy by itself or complementary to traditional treatments, such as surgery, chemotherapy, and radiotherapy [12].

At this moment, largely the TNM classification is used for staging and prediction in breast cancer patients. However, this classification has limited prognostic and predictive information, leaving room for an improved classification system [13]. Other factors that are important in the decision of treatment are grade, estrogen receptor (ER), progesterone receptor (PR), and human epidermal growth factor receptor 2 (HER2) expression [14]. Ki-67, a proliferation index marker, is also used in the clinical setting, but the analytical validation of this marker remains a topic of discussion, and evidence focuses primarily on ER-positive/HER2-negative tumors [15–17].

There is an urgent need for new biomarkers that allow for prognostic and predictive information and help to understand the pathophysiology of the underlying disease, especially for TNBC patients. The ideal biomarker should be non-invasive, easily measurable, and inexpensive, and have a high sensitivity and specificity [18]. Due to the considerable heterogeneity in breast cancer, the prediction of tumor behavior is especially important but challenging. Biomarkers can also aid in choosing fitting therapeutic options for individual patients, by means of predicting treatment responses [19]. Both the tumor microenvironment and the immune system are new, deployable, and promising biomarkers.

One aspect of the TME that has led to the development of a new prognostic and predictive histological marker is the tumor-stroma ratio (TSR). This method was developed based on the amount of intra-tumoral stroma in primary colorectal adenocarcinoma [20]. Since then, studies have demonstrated the correlation of the TSR to survival outcomes, concluding that patients with stroma-high tumors generally have a worse disease-free survival (DFS) and overall survival (OS) compared to patients with a low stromal content in their tumor. These outcomes were observed not only in patients with colorectal cancer [21–27], esophageal cancer [28, 29], and most other epithelial malignancies, but also in patients with breast cancer [30–36] — especially in the TNBC subtype. Moreover, the TSR was studied in relation to the pathological complete response (pCR) in breast cancer patients: a previous study of Hagenaars et al. showed that patients with stroma-low tumors had a higher pCR rate compared to patients with stroma-high tumors, which suggest a better response to neoadjuvant therapy in patients with tumors with a low amount of stroma [37].

With regard to the immune system, cluster of differentiation (CD) 4 and CD8 lymphocytes are subgroups of the tumor-infiltrating lymphocytes (TILs), which may vary in frequency in different breast cancer subtypes. This is of important clinical relevance, since specific breast cancer phenotypes respond differently to treatment modalities [38]. Seventy-five percent of the TILs can be subdivided into CD-types, such as CD3 + , CD4 + (T_helper_), and CD8 + (T_cytotoxic_) [39]. The prognostic value of TILs has been reviewed for various cancer types, including breast cancer [40]. It is thought that TILs can be used to assess the immune response against the tumor, in both the tumor and the micro-environment. It has also been described that the diverse molecular subtypes interact uniquely with the immune system, affecting prognosis and treatment response [41].

Other types of immune cells used as a prognostic marker for breast cancer patients include Forkhead Box P3-positive lymphocytes (T_regs_), Natural Killer (NK) cells [39, 40, 42, 43], and macrophages [44]. CD68 and CD163, for example, are tumor-associated macrophage (TAMs) markers, which recognizes M1 and M2 type macrophages [45, 46]. Another important element of the tumor associated immune cells is programmed death ligand-1 (PD-L1), also called CD279. PD-L1 is expressed on the surface of certain T-lymphocytes [47]. Tumor cells can also show abnormal high PD-L1 expression, which inhibits antitumor immunity. Therefore, immunotherapy targeting PD-L1 antibodies is a relevant treatment modality for these types of tumors. However, this therapy is only applicable for a selected group of patients. For that reason, biomarkers to predict treatment response and personalized treatment options are needed to determine which patients might benefit from these specific often costly treatments [48]. The TNBC subtype generally has a higher number of TILs, in comparison to hormone receptor-positive tumors [41]. However, according to the current European Society for Medical Oncology (ESMO) guidelines, there are not yet well-defined TIL thresholds for clinical decision making for therapy [49].

In this overview, we focus on the most frequently mentioned CD biomarkers in breast cancer, particularly lymphocytes and other immune cells, which constitutes the majority of the TILs. Since the large amount of research conducted over the years, with regard to the prognostic and predictive value of TME-related biomarkers, we have clustered this knowledge into a review. Both TILs and TSR are compared, side by side, to provide an overview of these biomarkers and their prognostic value, and to assess their clinical implication for women with breast cancer worldwide.

## Methods

This systematic review was reported according to the Preferred Reporting Items for Systematic Reviews [50].

### Search strategy

The electronic bibliographic databases PubMed, COCHRANE Library, Embase, Emcare, and Web of Science were searched for studies published between January 1, 2015, and January 31, 2020 (for TSR), and February 14, 2020 (for CD markers). The data were updated on February 13, 2024 (CD markers) and February 14, 2024 (TSR). Key search terms included “Breast cancer,” “tumor stroma ratio,” “CD-markers,” and “prognosis/early detection/prediction.” The complete search strategy, including search terms, is stated in the Supplementary Material (Supplementary Table 1). Search results were deduplicated in Endnote (Clarivate Analytics, Philadelphia, PA, USA).

### Selection criteria

Two authors (LA, SCH) independently evaluated the references as identified by the search and the eligibility of the articles based on title and abstract, followed by full-text assessments (Supplementary Figs. 1 and 2). Discrepancies were solved by consensus or by consulting a third author if needed (WEM). Original articles, focusing on patients with invasive breast cancer from all over the world, including randomized controlled clinical trials, case–control, and cohort studies, that were written in English or Dutch were considered for inclusion. During the final selection in February 2024, only articles that included results on TNBC were included, due to the high clinical need for the improvement of treatment selection in this specific subgroup and the large amount of studies in all subtypes together.

Exclusion criteria were a publication date before 2015, and the absence of an abstract or full text and animal studies, as well as meta-analyses, reviews, editorials, and case reports. Only data of papers focusing on histology and proteins’ expression (immunohistochemistry) were included. Studies focusing on the expression of mRNA/DNA or markers in serum were excluded. Patients with ductal carcinoma in situ (DCIS) were excluded from this review as well.

Furthermore, the most often mentioned immune markers in our search strategy were selected, to focus on markers which were most frequently analyzed in published studies, to cluster the available data.

### Outcomes

The primary endpoint of this review was survival rate. We focused on survival outcomes and prognostic information, such as represented in Kaplan–Meier curves (log rank analysis), and univariable and multivariable analyses, preferably mentioning hazard ratios (HR). Survival outcomes were compared of patients in groups with absent or low expression of specific markers to the outcomes of patients in groups with positive or high expression of markers.

### Data extraction

Data on study characteristics, entailing first author, year of publication, breast cancer type, number of patients, type of CD marker, and univariable and multivariable survival outcomes, were independently extracted from the included studies by LA and SCH. Both positive and negative study results were reported. Due to expected heterogeneity of the included studies and CD markers, a meta-analysis was not considered feasible.

### Definitions and statistics

The included studies in this review used various definitions of survival rates and focused on diverse locations within the tumor, such as intra-tumoral and stromal expression. The survival rates that were mentioned in the investigated papers are overall survival (OS), (invasive) disease-free survival (iDFS), relapse- or recurrence-free survival (RFS), recurrence-free probability (RFP), progression-free survival (PFS), breast cancer-specific survival (BCSS), distant metastasis-free survival (DMFS), and cause-specific survival (CSS).

For the selected markers, most articles researched expression in the entire tumor, but not all studies specified this explicitly. In this review, the exact locations were specifically indicated of the studied markers if mentioned in the results. When lymphocytes were directly in contact with the tumor cells, with no interference of stroma, there was an intra-tumoral (i) expression. If the lymphocytes were in the stroma between the tumor cells and had no direct interaction with tumor cells, there was stromal (s) expression [51]. The articles were therefore divided into three groups, based on expression: intra-tumoral, stromal, and the entire tumor (intra-tumoral and stromal combined, or not specifically mentioned).

The diversity between studies made it challenging to present the results consistently. Survival results were divided in different sections: Kaplan–Meier analysis and the results of the survival estimates, and univariable and multivariable analyses. Results were mentioned regarding a high or positive expression of the marker, in comparison to low or negative expression. Forest plots for each location were presented for markers and studies which provided necessary data, such as HR and confidence intervals (Figs. 2, 3, 4, 5, 6, 7; Supplementary Fig. 3). Overall, data of univariable analyses was used, but in case only data of multivariable analyses was presented, this data was used for the forest plot.

## Tumor-stroma ratio assessment

**Fig. 1:**
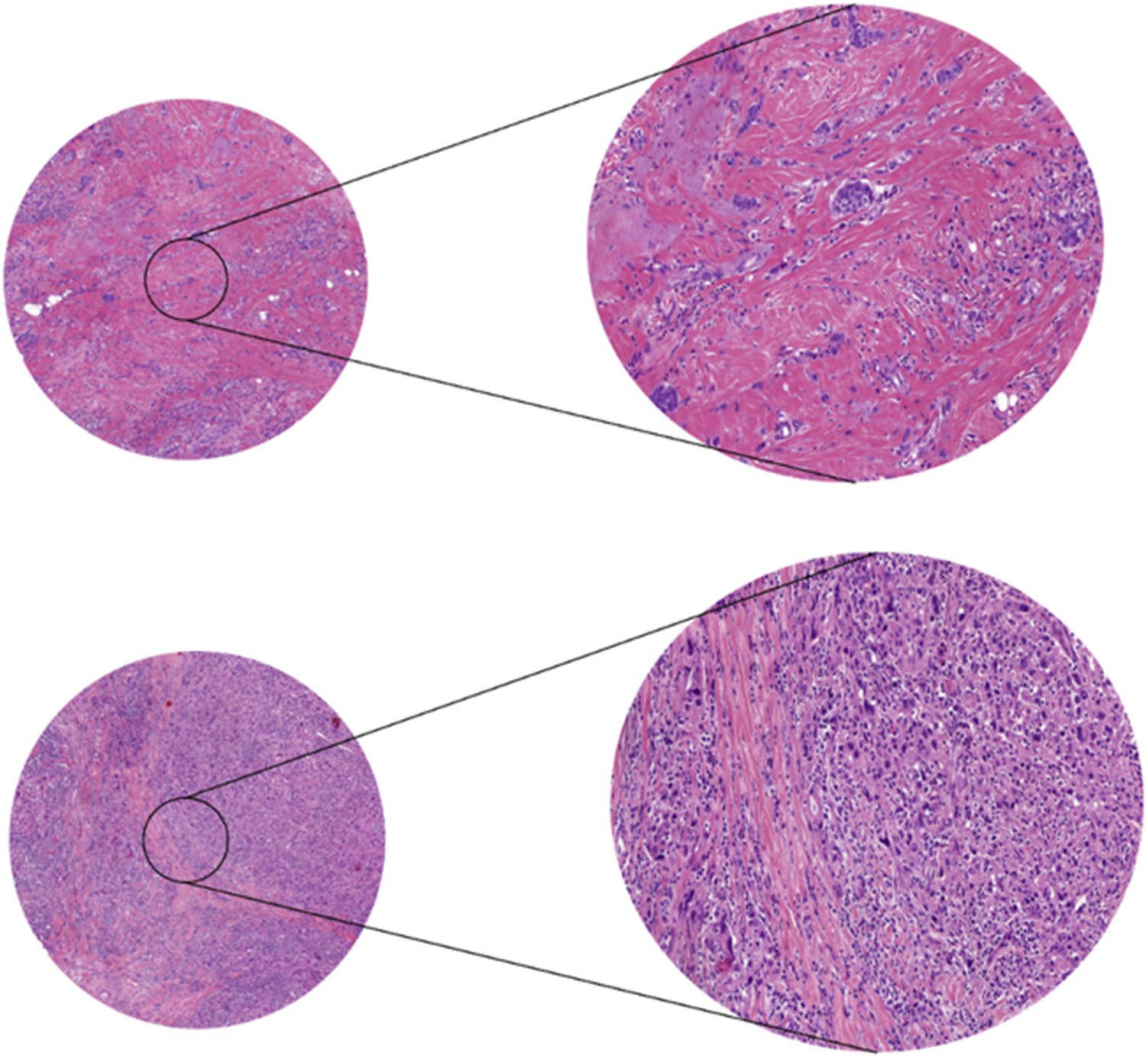
Example of stroma-high tumor (above) and stroma-low tumor (below)

Previously, Hagenaars et al*.* provided a thorough outline on the scoring method of the TSR in breast cancer patients [52]. Generally, scoring the TSR is performed on conventional hematoxylin and eosin (H&E)-stained slides, derived from biopsies or resection material of untreated patients. The slide with the highest amount of stroma should be chosen for scoring, using a conventional microscope or digitally on scanned tissue slides [52]. Scoring is done by visually estimating the amount of stroma compared to the tumor cells, in increments of 10%. To differentiate between stroma-high and stroma-low tumors (Fig. 1), a cutoff value of 50% is used. In case of > 50% stroma presence on the histological slide, it is classified as a stroma-high tumor. When the slide contains a stroma percentage of 50% or less, it is identified as a stroma-low tumor [20].

## Results: tumor-infiltrating immune cells and survival in triple-negative breast cancer

### Included markers

The most commonly described markers used to determine immune cells in recent years were included, such as CD3, CD4, CD8, CD24, CD44, CD68, CD163, FOXP3, and PD-L1, and combinations of these markers, namely CD4/CD8 and CD44/CD24 (Supplementary Table 2). These markers have played an important role in biomedical research for patient diagnosis over the last few years and are acknowledged as a novelty in the treatment of cancer [53]. In the included studies, immunohistochemical staining (IHC) was used for scoring of the TIL markers [52–56]. The number of cases with either low or high expression of a marker was generally spread evenly in the studies: this was the case since median expression levels often were used as a cutoff value to define a low or high expression.

A total of 43 unique articles investigating TILs in patients with TNBC were included in this review (Supplementary Fig. 1). Complete data of the included articles can be found in Supplementary Tables 3 – 13. Here, we present an overview of the associations between the expression of each marker and corresponding survival rates.

### CD8 expression

**Fig. 2:**
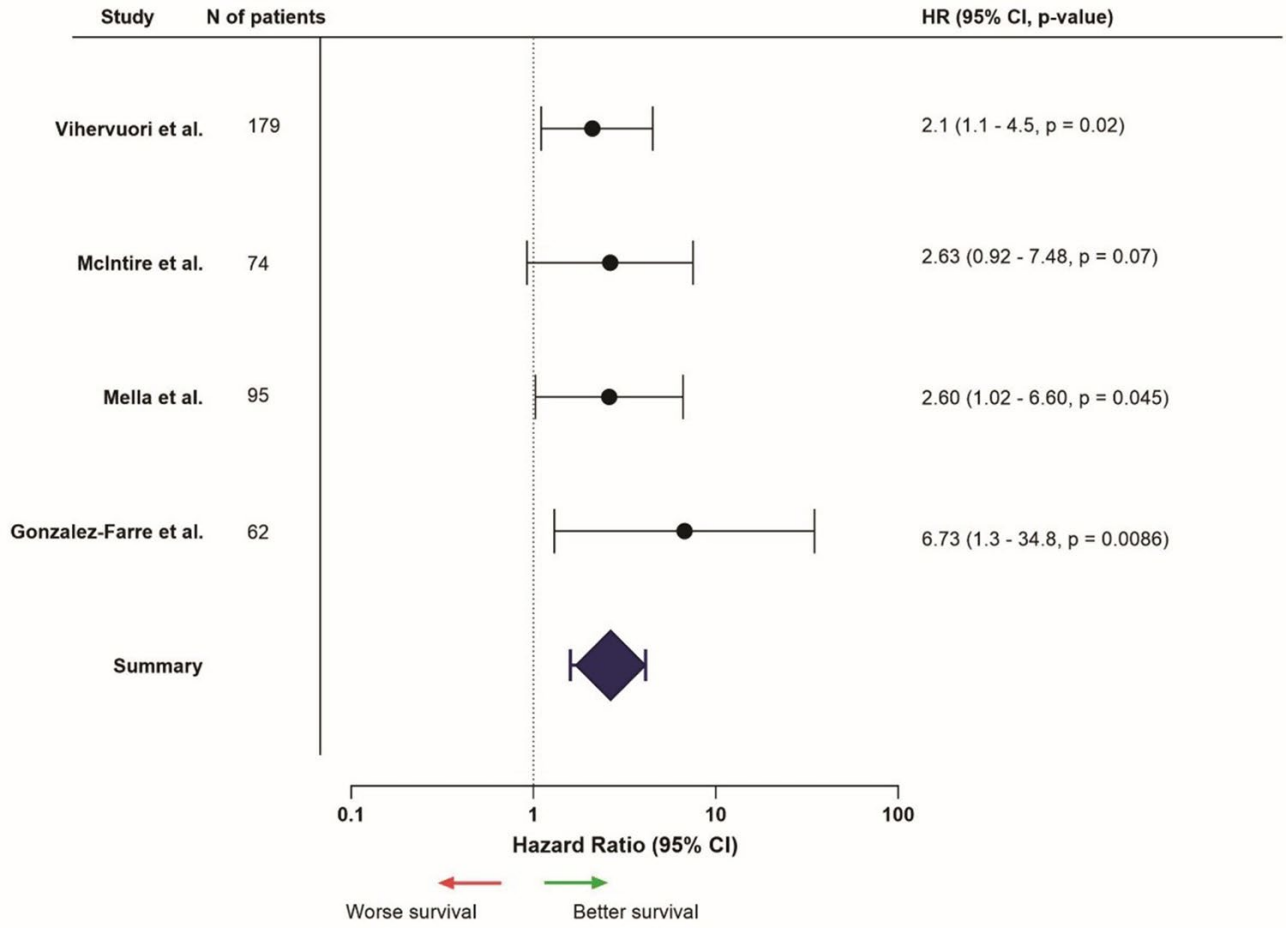
Hazard ratios (HR) related to high CD8 expression in the entire tumor (intra-tumoral and stromal, or undefined), HR > 1 = better survival, HR < 1 = worse survival (only presented for studies that provided HR, confidence interval, and *p*-value, from univariable analysis)

**Fig. 3:**
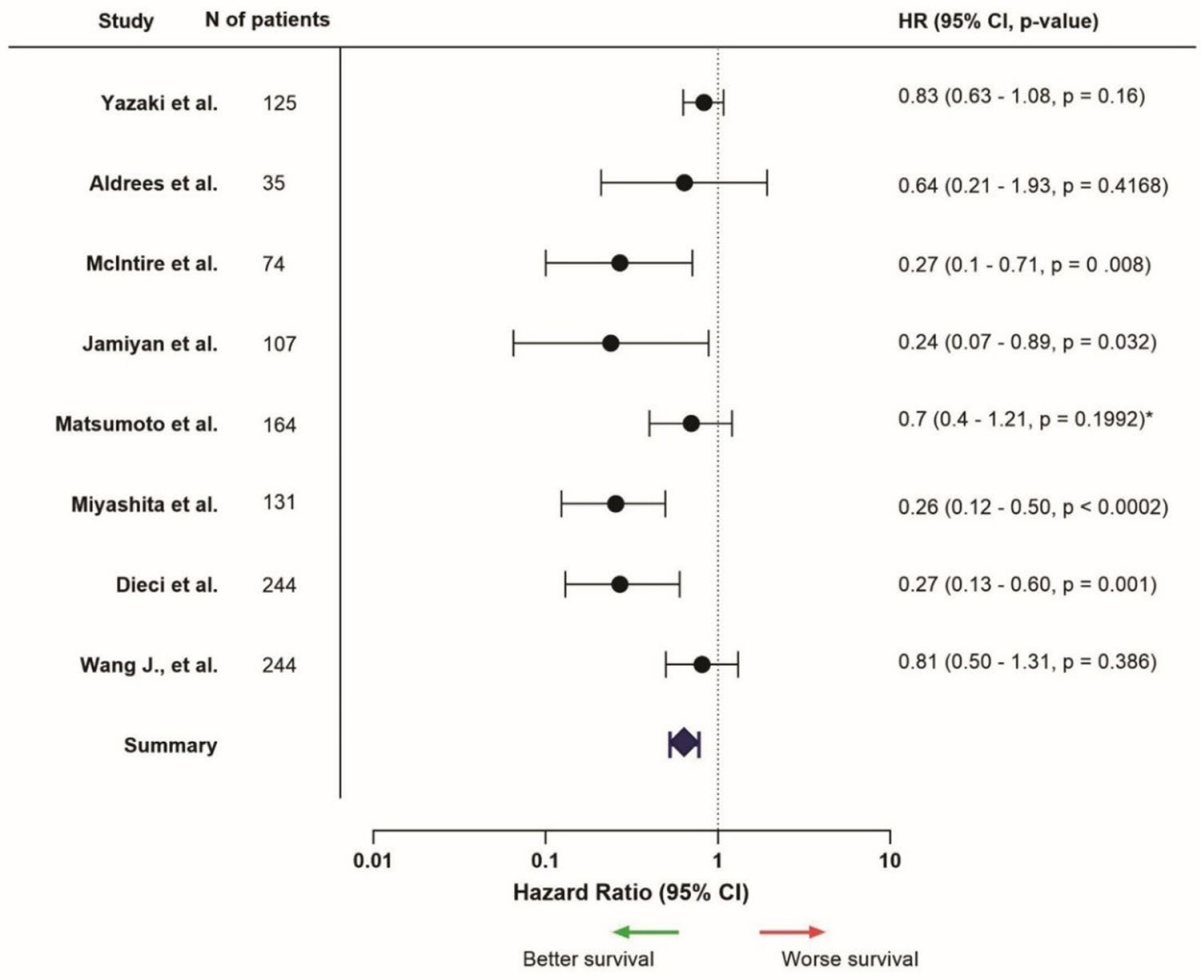
Hazard ratios related to high stromal CD8 expression, HR > 1 = worse survival, HR < 1 = better survival (only presented for studies that provided HR, confidence interval, and *p*-value, from univariable analysis). *Univariable data not available, multivariable data used instead

A total of 17 studies mentioned CD8 expression and survival results of TNBC (Supplementary Table 3). Five out of the six studies (83.3%) focusing on the entire tumor area found that high CD8 expression was associated with better survival outcomes [54–58]. One study (16.7%) found no survival difference between patients’ groups (Fig. 2) [59]. Intra-tumoral CD8 expression was described in seven studies: three out of the seven (42.9%) found better survival outcomes in patients with high expression [58, 60, 61]. Four studies (57.1%) showed no survival differences [62–65]. With regard to stromal CD8 expression, seven out of ten studies (70%) found more favorable outcomes related to CD8 expression [56, 60, 61, 66–69], whereas three studies (30%) did not report evident differences in survival analyses (Fig. 3) [59, 65, 70].

Overall, articles included in this review showed that high or positive CD8 expression in TNBC patients was associated with better survival rates, compared to low or negative CD8 expression.

### PD-L1 expression

**Fig. 4:**
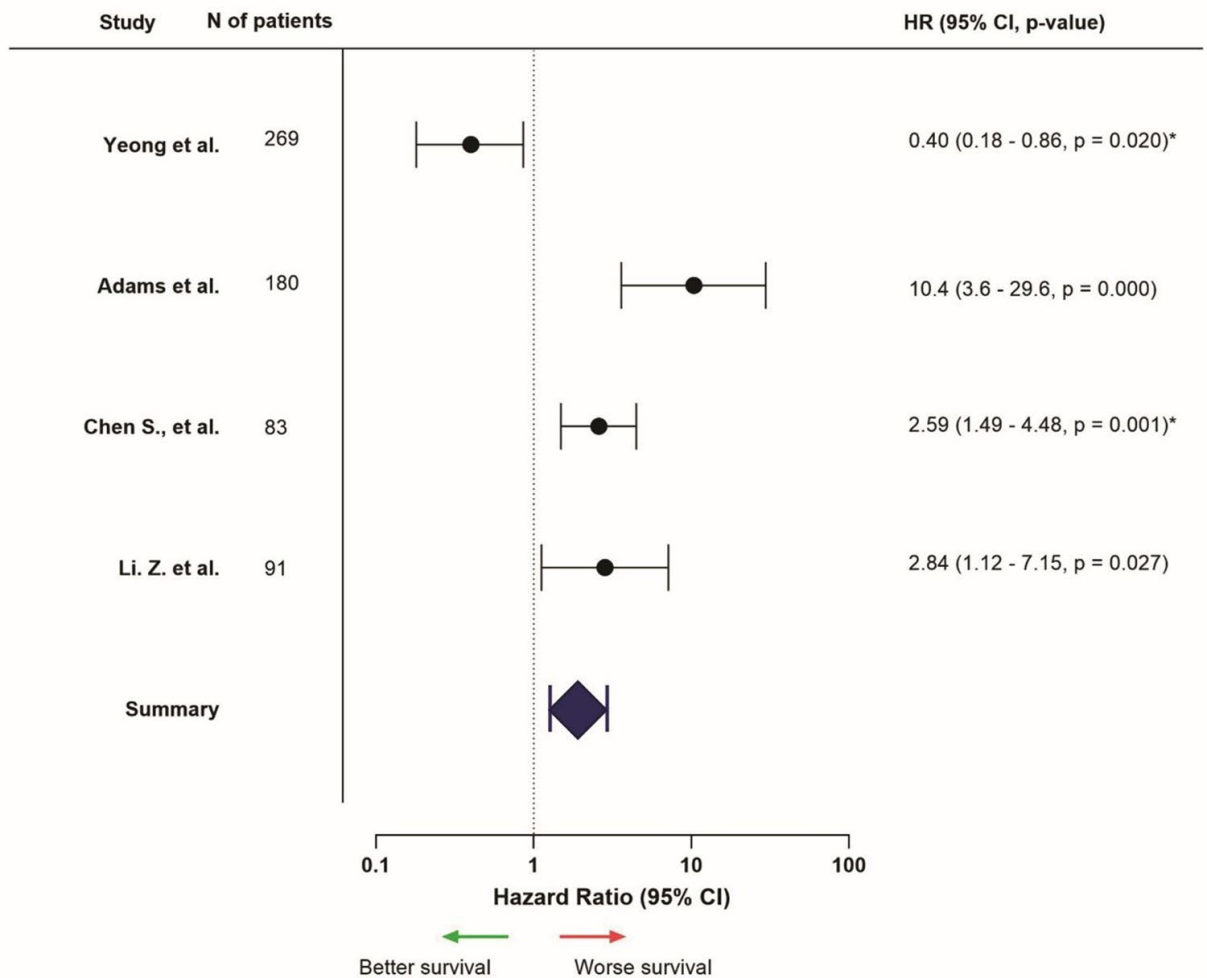
Hazard ratios related to high intra-tumoral PD-L1 expression, HR > 1 = worse survival, HR < 1 = better survival (only presented for studies that provided HR, confidence interval, and *p*-value, from univariable analysis). *Univariable data not available, multivariable data is used instead

**Fig. 5:**
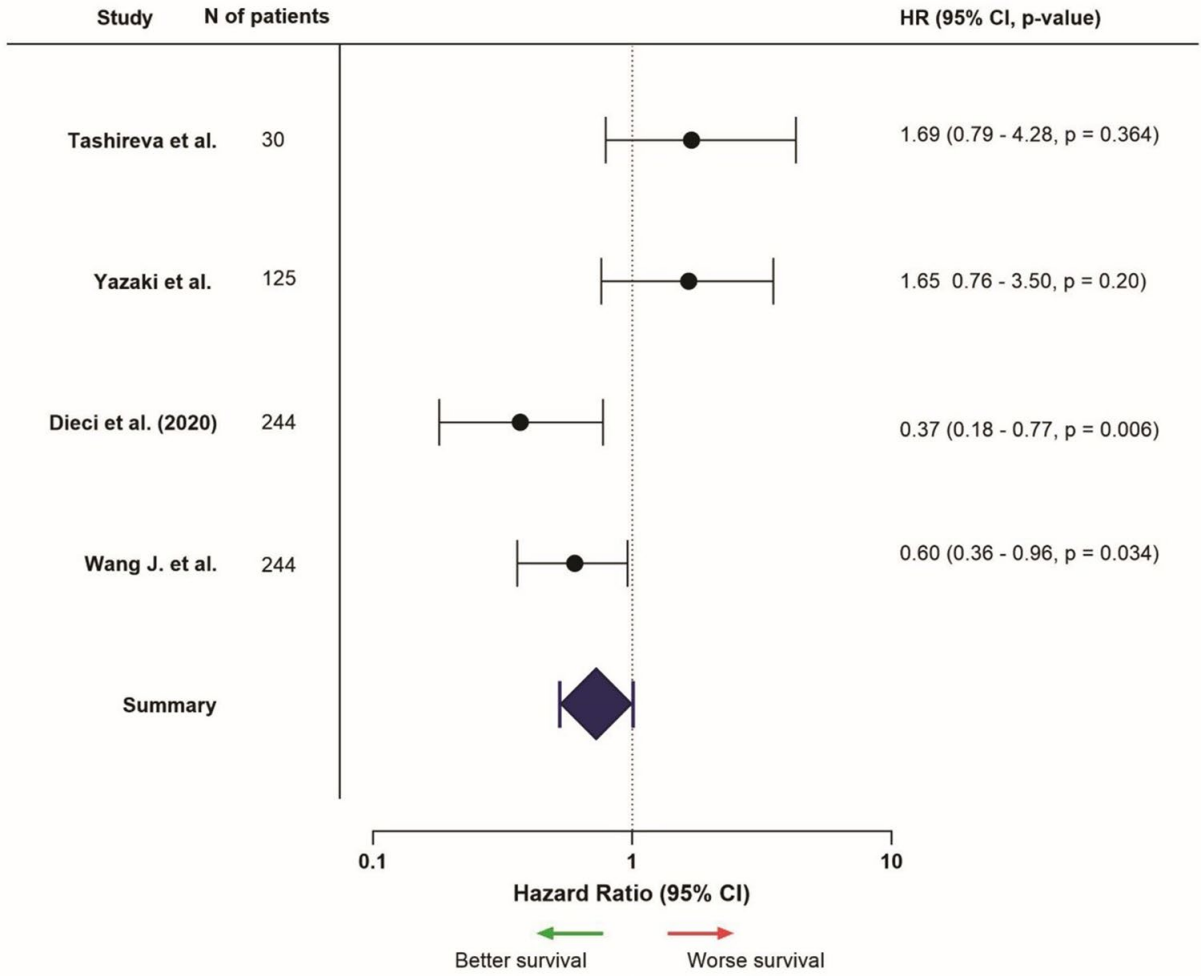
Hazard ratios related to high stromal PD-L1 expression, HR > 1 = worse survival, HR < 1 = better survival (only presented for studies that provided HR, confidence interval, and *p*-value, from univariable analysis)

Sixteen of the included articles mentioned survival results for TNBC patients regarding PD-L1 expression (Supplementary Table 4). One (25%) out of the four articles that mentioned PD-L1 expression in the entire tumor area showed a correlation with favorable survival [71]. The other three studies did not find any differences in outcome (75%) [63, 72, 73]. Five out of eight studies (62.5%) investigating intra-tumoral PD-L1 expression found poor survival rates in patients with high or positive expression of this marker [68, 74–77]. As opposed to these results, two studies (25%) found better outcomes when high or positive intra-tumoral PD-L1 expression was identified [78, 79]. One study (12.5%) found no different outcomes between patient groups (Fig. 4) [73]. Stromal PD-L1 expression was associated with improved survival outcomes in five out of ten studies (50%) [67, 68, 70, 80, 81], while one study (10%) found poorer outcome [69]. The four other studies (40%) found no correlation between stromal PD-L1 expression and survival (Fig. 5) [73, 78, 82, 83].

The results on PD-L1 expression in TNBC patients were opposite when focused on intra-tumoral expression compared to stromal expression. Regarding intra-tumoral expression, overall, worse survival outcomes were found. On the contrary, when focused on the tumor stroma, PD-L1 expression was correlated with more favorable outcomes.

### FOXP3 expression

In total, 15 studies mentioned FOXP3 expression and survival data on TNBC patients (Supplementary Table 5). In the one study (100%) that examined FOXP3 expression in the entire tumor area, there was a trend of better survival in patients with high FOXP3 expression [78].

**Fig. 6:**
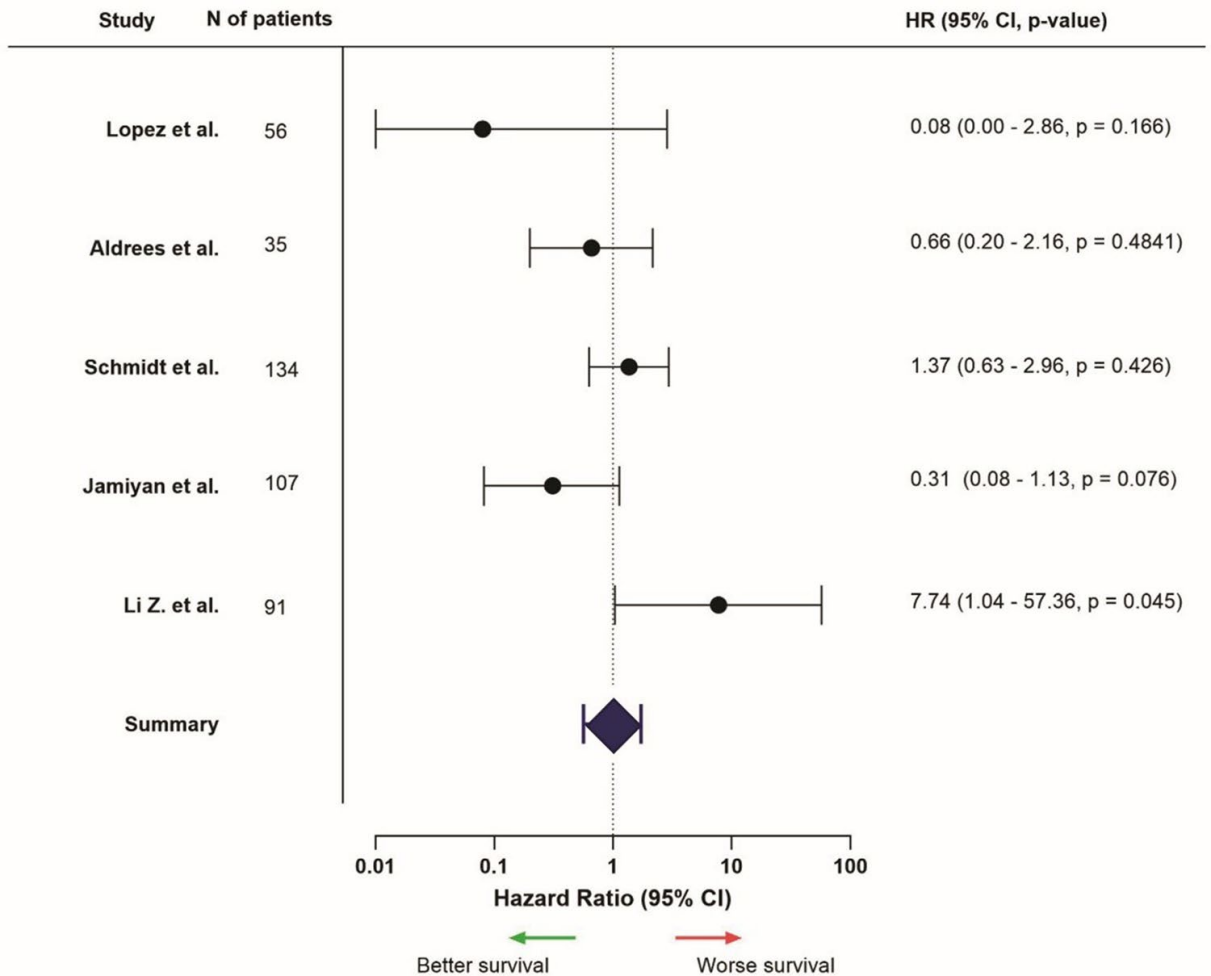
Hazard ratios related to high intra-tumoral FOXP3 expression, HR > 1 = worse survival, HR < 1 = better survival (only presented for studies that provided HR, confidence interval, and *p*-value, from univariable analysis)

**Fig. 7:**
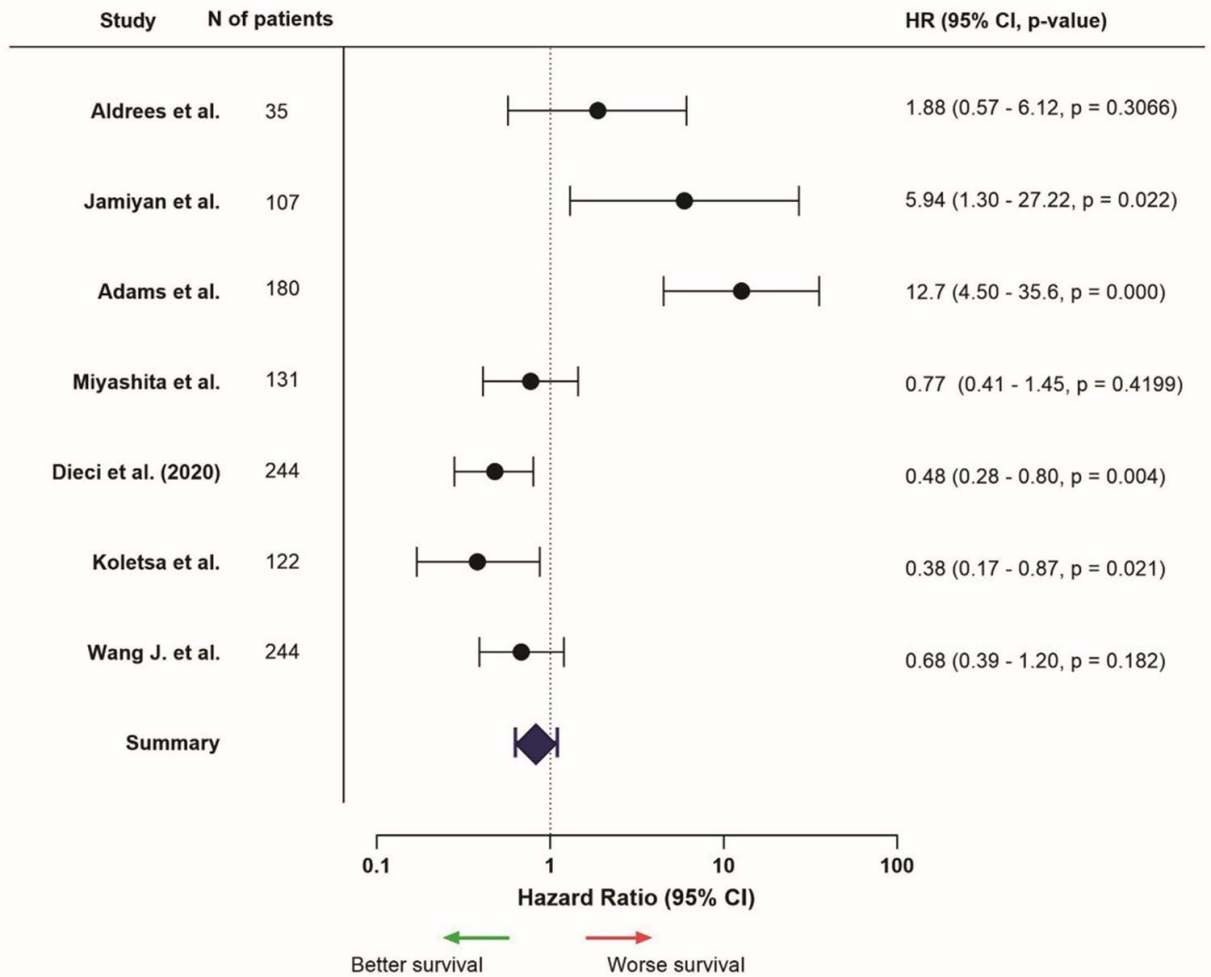
Hazard ratios related to high stromal FOXP3 expression, HR > 1 = worse survival, HR < 1 = better survival (only presented for studies that provided HR, confidence interval, and *p*-value, from univariable analysis)

Four (50%) out of eight studies examined intra-tumoral expression and found better outcomes in the high expression group [61, 62, 84, 85]. Two studies (25%) found worse outcomes [74, 86] and the remaining two studies (25%) showed no explicit relation with survival (Fig. 6) [65, 87]. Patients with high stromal FOXP3 expression had more favorable survival outcomes in six out of nine studies (66.7%) [66–68, 70, 84, 88]. Two studies (22.2%) found worse outcomes instead [61, 75], and one study (11.1%) did not find a relation with survival outcome (Fig. 7) [65].

In general, positive/high FOXP3 expression was associated with better survival rates in TNBC patients, especially when focused on its presence in the tumor stroma.

### CD3 expression

Four articles presented on survival results (Supplementary Table 6). One study (100%) presented results on CD3 expression in the entire tumor area, and found an association with better survival in patients with high expression [58]. Two out of three studies (66.7%) that focused on intra-tumoral expression found that patients with high CD3 expression had better survival rates [58, 89]. The third study (33.3%) did not find a difference in patient outcome [86]. Stromal CD3 expression was examined in two studies: one (50%) found better survival outcome [89], and the other (50%) found no difference [70]. No studies found worse survival outcome related to high CD3 expression.

Overall, CD3 expression is marginally correlated with more favorable survival outcomes in TNBC patients.

### CD4 expression

Eight papers mentioned CD4 expression and survival results specifically on TNBC patients (Supplementary Table 7). In one study, the expression in the entire tumor area was investigated, and no significant correlation was found between survival and the amount of CD4 positive lymphocytes [59]. Intra-tumoral CD4 expression was presented in six studies: two out of the six (33.3%) found a better survival in patients with high CD4 expression [60, 61]. Four studies (66.7%) found no difference in patient outcome related to the marker [62–64, 87]. High CD4 expression in the stroma was related to better survival rates in two out of four studies (50%) [60, 68]. One study found a worse survival (25%) [61] and another (25%) found no difference in outcome [59].

Generally, high CD4 expression appeared to be correlated with better survival results in TNBC patients.

### CD4/CD8 expression and CD4/CD8 ratio

Five studies mentioned CD4/CD8 expression and survival results on TNBC (Supplementary Table 8). Two studies examined the CD4/CD8 ratio in the entire tumor area: one study found a better survival in patients with high CD4/CD8 ratio [59], but the other study found a worse survival correlated with high CD4/CD8 ratio instead [55]. Regarding intra-tumoral expression, the three studies (100%) that focused on this area found better survival rates concerning high CD4/CD8 expression [60] and high CD4/CD8 ratio [61, 64]. Stromal expression was related to improved outcomes in two out of three studies (66.7%), in patients with high CD4/CD8 expression [60] and with high CD4/CD8 ratio [59]. Instead, the third study (33.3%) found a trend of shorter survival in patients with high stromal CD4/CD8 ratio [61].

The overall survival showed that patients with TNBC tumors presenting high CD4/CD8 expression or high CD4/CD8 ratio had better survival, compared to patients whose tumors had low expression.

### CD68 expression

Six studies examined survival outcomes in relation to CD68 expression in TNBC patients (Supplementary Table 9). Of those studies, one study (100%) focused on expression in the entire tumor area and did not find any correlation between survival and expression of CD68 [90]. High intra-tumoral CD68 expression was found to be associated with better survival in two (66.7%) out of three studies that focused on the expression in the intra-tumoral compartment of TNBCs [62, 81]. The third study (33.3%) found no difference in outcome between patients with low or high intra-tumoral CD68 expression [91]. Regarding high stromal CD68 expression, two out of three studies (66.7%) presented better survival rates in patients with high expression [68, 81], whereas one study (33.3%) did not find any correlation [70].

TNBC patients with tumors with high CD68 expression had more favorable survival outcomes, in comparison to those with low expression.

### CD163 expression

Five articles reported CD163 expression and survival results (Supplementary Table 10). One study focused on expression in the entire tumor area, and found better survival related to high CD163 expression (100%) [90]. One out of two studies found a poorer survival related to intra-tumoral expression [81], whereas the other study found no difference in patient outcome [91]. CD163 expression in tumor stroma was investigated in three studies: two (66.7%) out of three found more favorable outcomes [68, 81], and the third (33.3%) reported shorter survival in patients whose tumors showed high stromal CD163 expression [75].

There was no evident association between survival outcomes and CD163 expression in TNBC patients. However, there was a marginal trend of more favorable survival in patients with tumors with high CD163 expression.

### CD44/CD24 expression

Three studies included TNBC patients and examined their survival in relation to CD44/CD24 expression (Supplementary Table 11). These studies focused on intra-tumoral expression and all found (100%) worse survival outcomes related to positive or high CD44/CD24 ratio (CD44^+or high^/CD24^−or low^) [92–94]. There was no data on CD44/CD24 expression in TNBC patients with special attention to tumor stroma nor in the entire tumor area.

Intra-tumoral CD44^+^/CD24^−^ expression was associated with worse survival outcomes in TNBC patients observed for this small patient group.

### CD24 expression

Two studies presented results on CD24 expression and survival in TNBC patients (Supplementary Table 12). One study mentioned expression in the entire tumor area, and concluded that CD24 expression was associated with worse survival [95]. Another study focused on intra-tumoral expression and found worse survival in patients with high CD24 expression [77]. There was no data on CD24 expression in the tumor stroma specifically in TNBC.

The two studies, which examined different areas in the tumors, confirm CD24 expression in TNBC correlating with worse survival outcome.

### CD44 expression

Only one study provided survival outcomes of TNBC related to CD44 expression (Supplementary Table 13). This study examined intra-tumoral CD44 expression in TNBC following neoadjuvant therapy and found worse survival in patients with high expression of CD44 [96]. No papers presented data on CD44 expression in the entire tumor area nor in the stroma in the subgroup of TNBCs.

The only study that reported results on CD44 expression in intra-tumoral lymphoid cells of TNBC cases showed shorter survival related to high intra-tumoral CD44 expression. It should be noted that this study focused on patients treated with neoadjuvant chemotherapy.

**Fig. 8:**
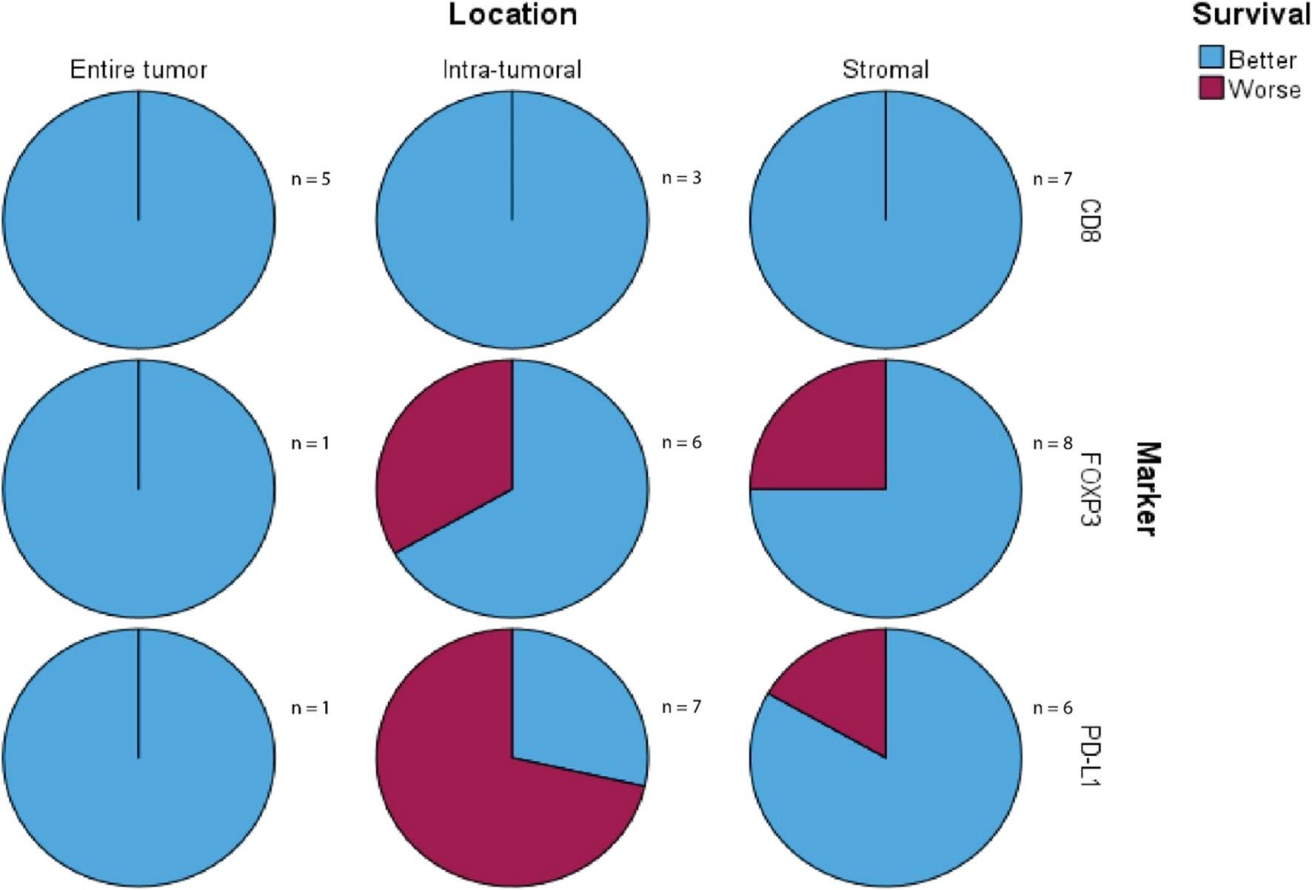
Overview of survival outcomes in TNBC patients, specified per marker and location: relation between high expression of a marker and survival (frequency of the results mentioned in this review is presented on the right side of the chart)

The graphical representation of the prognostic relevance of CD8, FOXP3, and PD-L1 in the different areas, based on the findings of our review, is shown in Fig. 8.

## Results: tumor-stroma ratio

Eight studies with results on the TSR in TNBC were derived from the literature search and included in this review (Supplementary Table 14). Tumors of patients included in the studies were most often classified as grade III and stage II tumors, mainly of the no special type. Almost all studies depicted a distribution in which approximately 60% of the cases had a stroma-low tumor and around 30% a stroma-high tumor.

**Fig. 9:**
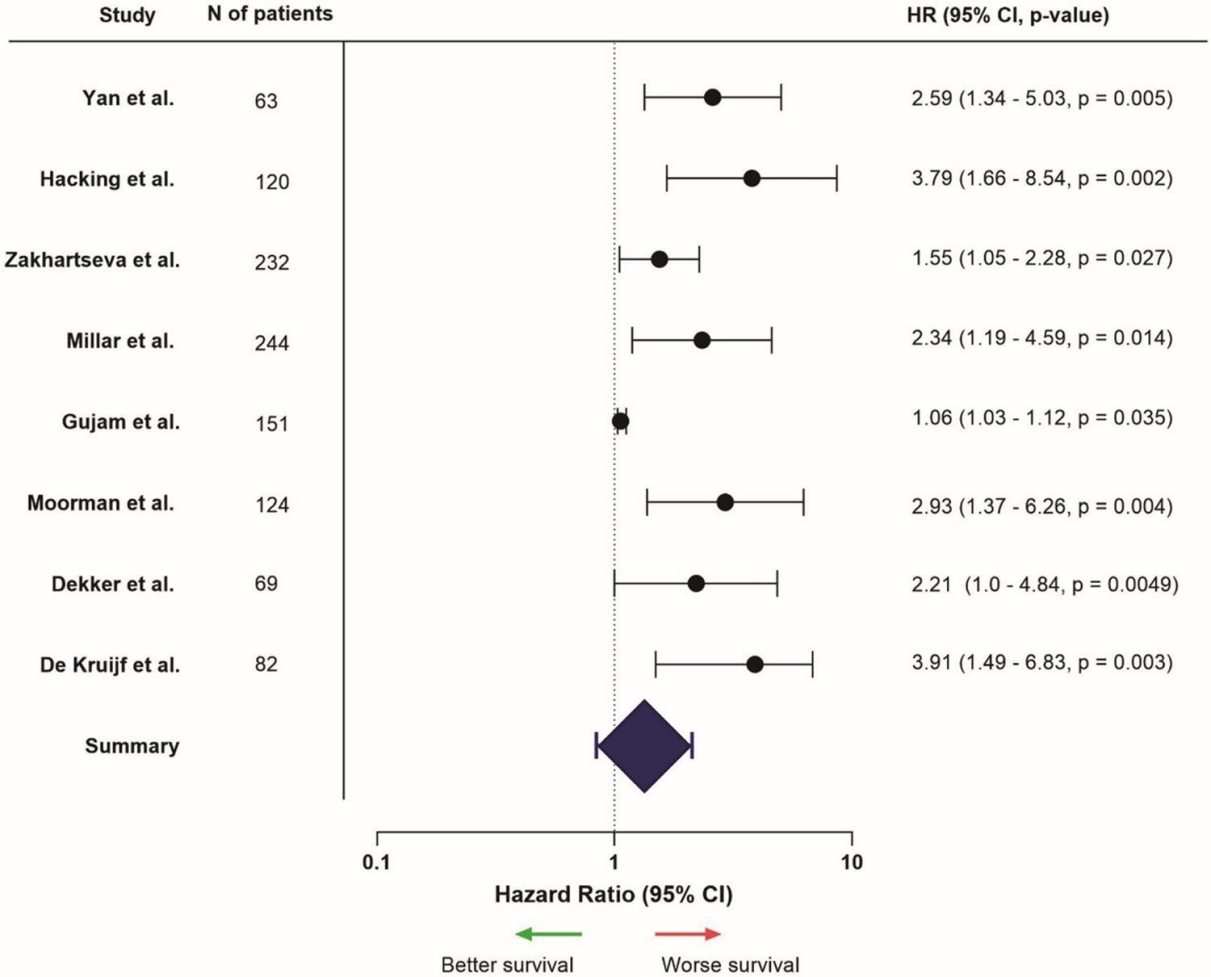
Hazard ratios related to high stromal content in in the primary tumor, HR > 1 = worse survival, HR < 1 = better survival (only presented for studies that provided HR, confidence interval, and *p*-value, from univariable analysis). *Hacking et al. examined patients treated with neoadjuvant chemotherapy specifically

All eight studies (100%) uniformly depicted worse survival outcomes in patients with a stroma-high tumor, compared to those with stroma-low tumors (Fig. 9). One of these studies focused on patients treated with neoadjuvant chemotherapy, but confirmed poorer outcomes in patients with stroma-high tumors [97].

In conclusion, high stromal content in in the primary tumor was associated with worse survival outcomes, especially in early-stage TNBC.

**Fig. 10:**
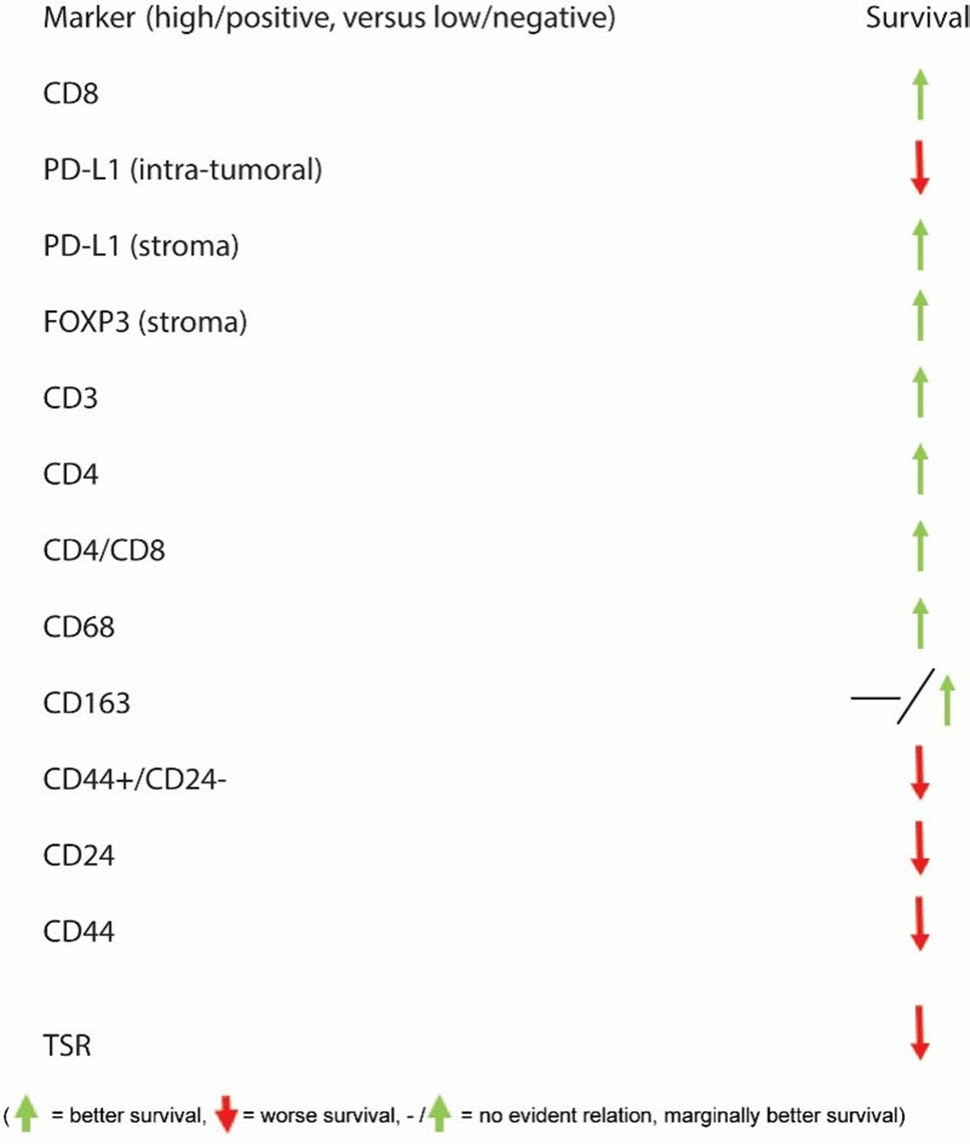
Overview of the relation between high expression of all markers and related survival outcomes

An overview of all included studies with survival outcomes in TNBC is provided in Fig. 10.

## Discussion

### Tumor-infiltrating immune cells

Translational research in patients with breast cancer has focused on many different markers from the TME and their possible prognostic values. The International TILs Working Group described that the added value of subtyping lymphocytes was not yet clear and therefore did not yet recommend the identification of specific subpopulations based on subtypes [98]. We aimed to analyze published studies that investigated specific subpopulations of TILs and their influence on survival outcomes to present a clear overview of the currently available knowledge on this topic.

In this paper, we reviewed 43 unique articles containing information on the subpopulation of TILs and other immune cells with correlating survival outcomes in primary triple-negative breast cancer patients. Generally, we found that a high number of CD8 + TILs was associated with better survival outcomes in TNBC. The presence of stromal FOXP3 expressing TILs was also associated with a more favorable survival. Other markers related to better survival outcomes, in case of positive or high expression, were CD3, CD4, CD4/CD8, CD68, and CD163, but there was only limited data available for these markers in relation to survival, specifically in the TNBC. Furthermore, it is important to highlight that some studies explain the ratios for CD4/CD8 differently. For example, Thian et al*.* defined a high CD4/CD8 ratio as high CD4 expression in relation to low CD8 expression, whereas Matsumoto et al. defined high CD4/CD8 expression as CD4-high and CD8-high expression [60, 64]. This makes it challenging to interpret the different outcomes consistently, next to the other causes of heterogeneity between the studies. TIL markers associated with shorter survival in TNBC were CD44^+^/CD24^−^, CD24^+^, and CD44^+^. However, this was again based on a restricted number of studies included in this review.

What stood out were the outcome data for PD-L1 that were not concordant with each other, based on the location of the expression. Patients with high stromal PD-L1 expression more often had favorable survival outcomes. Conversely, TNBC patients with high intra-tumoral PD-L1 expression had unfavorable survival rates, in comparison to those with low intra-tumoral expression. The discrepancy could be due to several reasons. PD-L1 is important in the immune response, which might be disrupted in carcinogenesis. High presence of PD-L1 positive T cells in the stroma may reflect an active immune response and better defense mechanism against the cancer, which results in better survival outcomes. Another possibility is that tumors with high stromal expression are more sensitive to immunotherapy, which was not addressed in this review [99–101]. On the other hand, intra-tumoral PD-L1 expression might reflect a more aggressive carcinoma since increased PD-L1 expression also results in immune evasion and immunosuppression [102–104].

The discrepancy between intra-tumoral and stromal PD-L1 expression again underlines the prominent role of the TME in cancer development. Heterogeneity in tumor genetics, and also the area on which the expression of markers, is scored might influence not only survival, but also the interpretation of the results. Another possible explanation is the heterogeneity in patient characteristics, such as age and ethnicity, which makes patients with a specific subtype of breast cancer like TNBC a diverse group. Besides that, different study designs and methodology, such as sample sizes and used cutoff values, could also influence outcome.

A previous meta-analysis of Li et al. also showed that a high CD8 expression is associated with better survival in cancer patients treated with immunotherapy, such as melanoma, non-small cell lung cancer, and breast cancer, which shows the clinical importance of the use of CD markers in the treatment of cancer [105]. Various studies already mentioned a correlation between TILs and recurrence, DFS, and OS in breast cancer patients [43, 106]. A previous meta-analysis of Wang et al*.* mentioned that high TIL levels were associated with significantly better DFS and OS in breast cancer patients. They concluded that high TIL levels, mainly characterized by CD8-positive lymphocytes, were a strong prognostic factor, which was in line with the data in this review [43].

There were also a number of results that were not in line with each other. For example, a meta-analysis of Guo et al*.* found that patients with tumors with positive or high PD-L1 expression had worse outcomes, compared with those with tumors showing negative or low expression [107]. These results were not supported by the findings with other reports in our review, where we found an association with better survival instead. However, it is important to note the importance of different breast cancer subtypes and the location of immune cells within the tumor. In the meta-analysis of Guo et al., there was no evident distinguishment between the different molecular subtypes and whether PD-L1 expression was examined in the entire tumor area, intra-tumoral, or in stromal compartments [107].

Earlier research also showed poorer survival outcomes in relation to FOXP3 expression [74, 86, 108], whereas in this review there generally was an association with better survival [67, 68, 84, 88]. It is important to mention that when focusing on significant results only, there was already a discordance between survival outcomes. Here, worse outcomes were mainly found in studies focused on intra-tumoral FOXP3 expression, in all breast cancer subtypes [74, 86, 108]. In this review, which focused on TNBC patients, we generally found a more favorable outcome, but specifically related to stromal FOXP3 expression [67, 68, 84, 88]. This again underlines the importance of regarding the tumor and TME as a whole, instead of only focusing on one compartment.

The International TILs Working Group advices to focus on the stromal compartment, instead of the intra-tumoral TILs (iTILs) or the entire tumor area. This was recommended because the stromal TIL (sTILs) count is less influenced by the expansion pattern and density of tumor nests since stromal TILs are measured in between the tumor nests instead. Furthermore, iTILs are more challenging to score without immunofluorescence and immunohistochemistry, and are generally present in smaller amounts and in fewer cases [98]. In our review, we differentiated between the location of the lymphocytes’ subtypes and, furthermore, focused on results for TNBC. The different survival outcomes between intra-tumoral and stromal PD-L1 expression not only emphasize the important role of the tumor-environment in tumor progression, but they also show why TME subgrouping is important for personalized treatment. Thus, the location of the marker is of importance, along with attentiveness for the heterogeneity between and in the different breast cancer subtypes.

### Implementation in clinic

Since there is a great diversity in outcomes among breast cancer patients, the use of prognostic markers might be of clinical relevance to help in personalizing treatment [109]. The diversity emphasizes the importance of using these markers correctly. PD-L1 has previously been used as a predictive marker of response to immunotherapy in other cancer types, such as non-small cell lung cancer [110]. Biomarkers could aid in selecting patients who might benefit from immunotherapy [111–113]. Tools, such as MammaPrint and Oncotype DX, aid in the decision if systemic therapy could be omitted or not [114, 115], but are also mainly focused on ER-positive patients and early-stage breast cancer [116], and they might not be accessible for every patient [117–119]. This form of individualized therapy is especially relevant for TNBC because of its aggressive nature and limited treatment possibilities.

Previous studies suggested that TNBC contains higher levels of TILs, which might make this subtype more susceptible to immunotherapy [101, 120, 121]. PD-L1 is thought to be expressed in about 20% of TNBC and in vivo research suggested that tumors with high PD-L1 expression had a better response to immune checkpoint inhibitor therapy [122–124]. However, the ESMO guidelines do not (yet) advice to use PD-L1 expression levels to guide therapy choices in early breast cancer [49], and suggest further research in this field. Moreover, it is important that several subpopulations of TILs are thought to influence each other. Adams et al*.* found a high-level correlation between CD163, PD-L1, and FOXP3 and suggest that a combined therapy, focused on PD-L1 and regulatory T-cells, is perhaps more suitable [75]. The use of TILs in a clinical setting could thus be of great consequence. However, as shown in this review, there is a great variety between outcome rates in the presence of specific subgroups of lymphoid cells in TILs. Therefore, there is a risk in only using TILs as a whole, because as shown here, different markers are correlated with different survival outcomes even in the single subgroup of TNBC.

### Limitations

In this review, an important limitation are the small study samples. Another important limitation was the use of different research methods as was earlier mentioned: some articles focused on TIL infiltration only in the tumor center, others in tumor stroma. It was also not always described whether the expression was scored based on intra-tumoral infiltration or stromal infiltration, or both. Furthermore, different cutoff values were used for low or high expression of the markers, and for absence or present expression. This makes scoring TILs especially challenging, and even more so to compare different studies with each other.

This limitation was especially relevant for PD-L1 expression, due to the different possible antibodies to score PD-L1 positivity in practice: with SP142, the immune cell positivity in stroma is scored and a score of > 1% is defined as a positive case. With antibody 22C3, the Combined Positivity Score (CPS) is identified, where the positive tumor cells and positive immune cells together are divided by the number of all the tumor cells (multiplied by 100). A value > 10 in this case is defined as positive [125, 126]. Most of the studies focusing on PD-L1 in our review used SP142 as an antibody [63, 69, 80, 81, 83], and another used 22C3 [78], but in other cases, different antibodies were used, such as Abcam (ab58810 [74], or ab205921 [76]) or SP263 [73]. Furthermore, there is a different use of terminology, which make the results challenging to interpret the findings uniformly. According to the TILs working group, intra-tumoral TILs expression is defined as a direct interaction with tumor cells [127]. However, the included papers appear to use the definitions (expression on tumor cells only, and on inflammatory cells) interchangeably. In the research on TNBC, SP142 is most commonly used, as described above. However, previous studies showed a discrepancy between SP142 and 22C3 regarding PD-L1 positivity, and results in Miyakoshi et al*.* suggest that the combination of SP142 (immune cells) and 22C3 (CPS) results in a higher concordance in TNBC [125, 128]. Our advice would thus be to follow the definitions and recommendations of the International TILs Working Group, as to create consensus, and focus on both tumor cells and immune cells, due to the substantial role of the micro-environment.

The TILS Working Group earlier published a paper on their recommendations on the scoring method to improve reproducibility [98]. They advise focusing on the stromal TILs, within the border of the invasive tumor, to include all mononuclear cells and not to focus on hotspots. The assessment of TILs was performed according to this guideline by several of the included papers on CD8 [54, 56, 58, 63, 66–70]: the application of these suggestions in the studies might have contributed in the reproducibility of the results on CD8 in this review.

In addition, the survival outcomes were presented differently, which makes it challenging to compare results between included studies and perform a forest plot for each marker. Furthermore, not all studies presented results for both univariable and multivariable analyses, which resulted in forest plots merged with different type of analyses.

Future studies should include a large study population, with survival outcomes for each different subtype of breast cancer. Furthermore, there should be a focus on the subpopulations of lymphoid cells that are part of TILs, with more attention to the expression in different compartments, such as intra-tumoral and stromal expression. With regard to clinical relevance, future studies should focus on combining CD expression with treatment modalities, so it can be used as a prognostic marker for survival outcomes and predict survival outcomes in specific groups of patients. However, studies regarding the selection of patients for immunotherapy should take into consideration that patients with high expression of several markers (CD8, CD3, stromal PD-L1) have shown to have a more favorable prognosis already, compared to low expression, as is shown in this review. Therefore, there is a risk of confounding in studies that show better survival outcomes in, for example, patients with high PD-L1 expression with more favorable survival outcomes after receiving immunotherapy, compared to those with low PD-L1 expression [121].

The heterogeneity of breast cancer, due to both tumor and patient characteristics, is important to consider in future studies, to make individual and effective treatment possible.

### Tumor-stroma ratio

We included eight unique articles focusing on the tumor-stroma ratio and found a correlation between stroma-high tumors and poorer survival rates in TNBC, compared to patients with stroma-low tumors. A previous review by Kramer et al. already presented an overview of the prognostic value of the TSR in TNBC specifically, in 2019 [129]. This study also addressed the importance of further exploration of the TSR, particularly in this subgroup. There are limited effective therapy options yet and the aggressive nature of this subtype makes the clinical need for studies in this particular group high [129]. An example of using the TSR in combination with therapy by Hagenaars et al. showed that the TSR was associated with response to neoadjuvant chemotherapy: stroma-low tumors were significantly correlated with better Miller-Payne scores and better pathological responses, compared to stroma-high tumors. This shows the potential of the TSR in predicting which patients will react to neoadjuvant chemotherapy, to help in personalizing therapy and preventing undertreatment and overtreatment [37].

An advantage of the TSR, compared to the TILs as a whole, is that it takes both the tumor and the tumor-microenvironment in consideration in the process of prognostication. Another advantage of using the TSR is that the process of scoring is quick, relatively cheap, and easy: there is no extra staining needed and conventional H&E-stained slides of resection material or biopsies can be used [52]. This is in contrast to using the TILs’ subpopulations for prognostication, in which at least additional immunostaining is needed to examine various cellular components of TILs, such as for CD8 and CD3. Another matter to take into consideration is the difference in techniques used for scoring the subtypes of the TILs, such as the used variety in thresholds and the methods of staining, whereas assessment of the TSR is done more evenly. As was provided in the review by Kramer et al*.*, the interobserver variability for scoring the TSR in studies focusing on TNBC was between 0.68 and 0.85 [129]. The interobserver agreement for TILs is harder to examine, since there are often different subtypes investigated. A study of Swisher et al*.* found a kappa of 0.57 for sTILs and 0.65 for iTILs in TNBC and Tramm et al*.* found kappa values ranging between 0.38 and 0.46 for sTILs in a study with all types of breast cancer [130, 131]. O’Loughlin et al*.* assessed both the interobserver and intra-observer agreement in TNBC [132]: a kappa of 0.53 was found for intra-observer agreement when assessing sTILs in increments of 25%, and 0.24 for increments of 10%. Interobserver agreement was expressed in intraclass correlation coefficient (ICC), which was 0.595 for scoring sTILs. This shows that the TSR is both easier and more reproducible to score, specifically in TNBC.

At the moment, the results of the TSR in different studies are generally more consistent and in line with each other. This is in contrast to the CD markers, of which previous literature shows a wide variety of outcomes, as was shown in this review. A previous article of Ocana et al*.* (2015) also presented an overview of TILs in breast cancer: they found that stromal TILs are associated with better survival in TNBC, whereas intra-tumoral TILs show more heterogeneous results [133]. They concluded that TILs as a prognostic marker was not yet ready to be used, due to not having a standardized approach which has been validated in multiple settings [133]. With the data we clustered here, from data published in 2015 and after, it seems there is still not yet a standardized method which has consistently been validated.

Overall, this seems to make implementation of the TSR more feasible at the current moment. Moreover, recent research of our group shows that adding the TSR to the already implemented PREDICT model [134] — an online prognostication tool to aid in clinical decision making — leads to improvement of the model, especially in the TNBC subgroup (data not published yet) [135].

However, further research and validation of its predictive potential is still needed, before actual implementation of the TSR in clinical setting is possible in breast cancer, since the (added) value of the TSR in immunotherapy and endocrine therapy is still unknown and a relevant subject to investigate in future studies.

It would also be interesting to combine the markers, as was done by Ravensbergen et al*.* They combined TSR and tumor-infiltrating immune cells (TIIC) to predict the response to immune therapy in colon cancer and found that stroma-low/immune-high tumors were most responsive to immune checkpoint inhibitor therapy [136]. Therefore, both the TSR and TILs as biomarkers could improve the prognostication of patients and give more insight into the prediction of treatment outcomes of breast cancer patients [20, 33, 137]. In another study, by Li et al*.*, the combination of the TSR and stromal TILs was a promising predictor of pCR, especially in HER2-negative patients [138]. Furthermore, Vangangelt et al*.* described that combining immune status with TSR resulted in a stronger predictive value of the TSR [34]. These studies have made a start in elucidating the potential of uniting these two parameters. The TSR might play a role in the prediction of the aggressiveness of the tumor and possible survival outcomes, and the TILs could be used to predict the response of the immune system in the process of tumor progression. This way, both markers might strengthen each other and be of even more significance. This might be of relevance, especially for patients with TNBC, with attention to the relatively low survival rates and high recurrence rates [139, 140]. In this group, the abovementioned markers could aid in the prognostication and targeting of disease and offer patients individualized, fitting treatment to improve their survival outcomes.

## Conclusion

To individualize the treatment of triple-negative breast cancer patients, we recommend to use a predictive biomarker focusing on a subpopulation of the TILs, such as CD8, which has shown to provide more consistent results in the TNBC subgroup in previous studies. We particularly think the marker PD-L1 is of interest because of the contrasting results when focused on intra-tumoral expression, against stromal expression, which should be further examined. This underlines the importance of involving the microenvironment in the process of personalizing treatment of breast cancer.

Our review supports earlier findings by our group that the TSR has the potential to be a substantial prognostic marker: high stromal content is repeatedly correlated with worse survival, especially in the TNBC subtype. A start is already made in using the TSR to predict which patients could benefit from treatment, by researching the correlation between stroma and treatment responses.

With this review, we present an overview in the usage of subpopulations of TILs and TSR in the prognostication of breast cancer patients and underline the important role of the tumor-microenvironment in cancer progression. Future research should focus on a combination of both markers and on therapy-related outcomes, to aid in choosing the appropriate treatment regimen of women with TNBC in particular.

## Supplementary Information

Below is the link to the electronic supplementary material.

Supplementary file1 (DOCX 398 KB)

## Data Availability

Data sharing is not applicable to this article as no new data were created or analyzed in this study. Therefore, the findings of this study are available within the article and its supplementary materials.
