# Supplementary material for: The prognostic value of the tumor-stroma ratio compared to tumor-infiltrating lymphocytes in triple-negative breast cancer: a review
Source: Virchows Arch. 2025 Feb 4;486(3):427–44. doi: 10.1007/s00428-025-04039-z (PMC11950021; doi:10.1007/s00428-025-04039-z)
Supplement: Supplementary file 1 — Supplementary file1 (DOCX 398 KB) [file 428_2025_4039_MOESM1_ESM.docx]

**Supplementary data**

Supplementary Figure 1: Flowchart from included articles on TILs and TNBC

**Identification of studies via databases and registers**

Records identified from:

Databases (n = 2196)

Records removed before screening:

Duplicate records removed (n = 8)

**Identification**

Records excluded (n = 810):

Meeting abstracts (n = 790)

Published before 2015 (n = 20)

Records screened

(n = 2188)

Reports not retrieved (n = 10):

No abstract, full text not relevant (n = 8)

Article no full text available (n = 3)

Reports sought for retrieval

(n = 1378)

**Screening**

Reports excluded:

Based on title (n = 1018)

Based on abstract (n = 128)

Based on full text (n = 93)

Based on top-10 markers (n = 50)

Based on no results on TNBC (n = 35)

Reports assessed for eligibility

(n = 1367)

Studies included in review n = 43

Supplementary Figure 2: Flowchart from included articles on TSR and TNBC

**Identification of studies via databases and registers**

Records identified from:

Databases (n = 450)

Records removed before screening:

Duplicate records removed (n = 176)

**Identification**

Records excluded (n = 30):

Published before 2010 (n = 30)

Records screened

(n = 391)

Reports not retrieved: n = 0

Reports sought for retrieval

(n = 361)

**Screening**

Reports excluded:

Based on title (n = 308)

Based on abstract (n = 20)

Based on full text (n = 12)

Based on no results on TNBC (n = 13)

Reports assessed for eligibility

(n = 361)

Studies included in review: n = 8


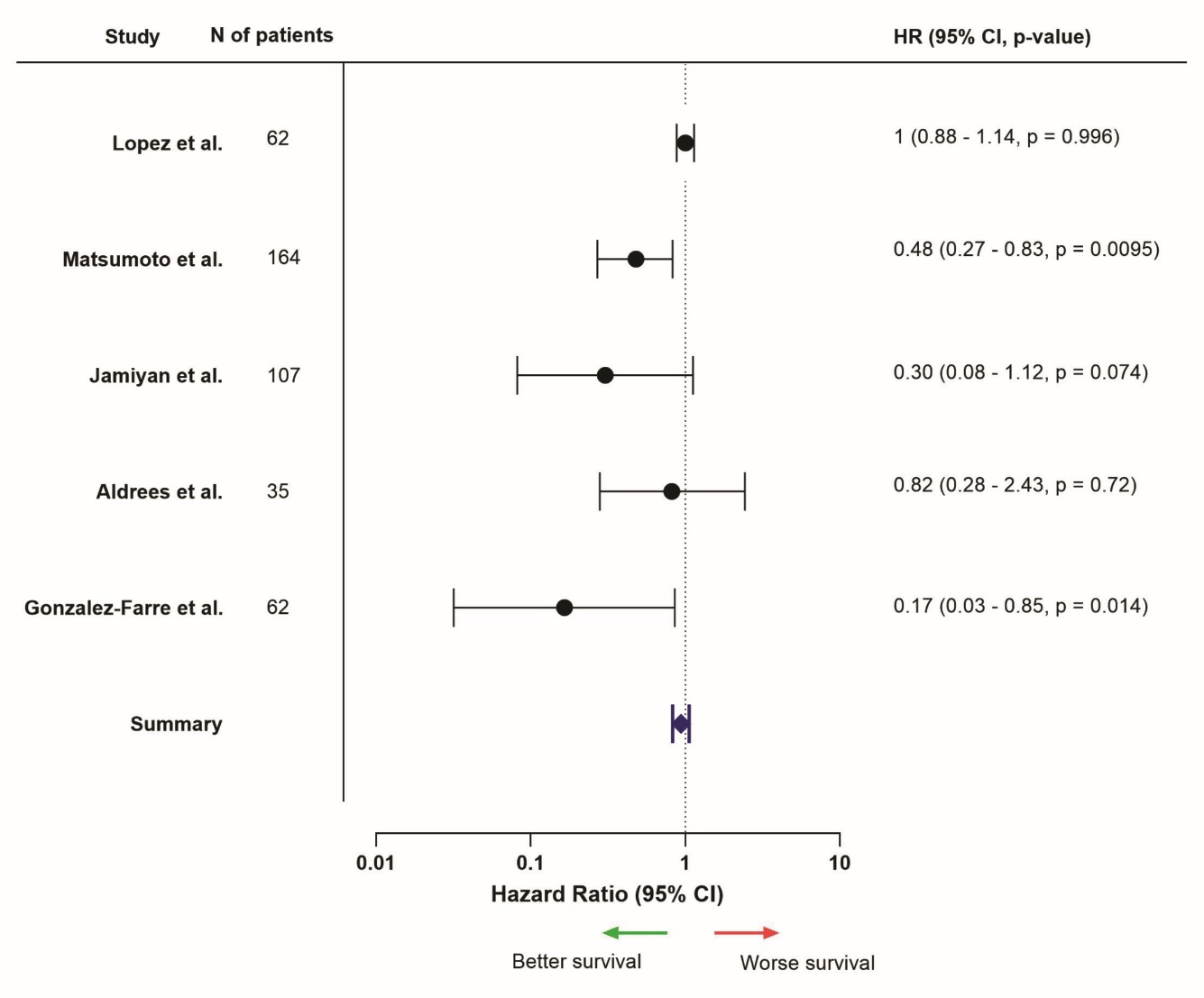
Supplementary Figure 3: Hazard ratio’s related to high intra-tumoral CD8 expression, HR>1 = worse survival, HR<1 = better survival (only provided for studies that provided HR, confidence interval and p-value)

Supplementary Table 1: search query as used in Pubmed

| Search query tumor infiltrating lymphocytes | (("Breast Neoplasms"[majr] OR "breast cancer"[ti] OR "breast cancers"[ti] OR "breast carcinoma"[ti] OR "breast carcinomas"[ti] OR "breast adenocarcinoma"[ti] OR "breast adenocarcinomas"[ti] OR "breast tumor"[ti] OR "breast tumors"[ti] OR "breast tumour"[ti] OR "breast tumours"[ti] OR "breast neoplasm"[ti] OR "breast neoplasms"[ti] OR "breast malignancy"[ti] OR "breast malignancies"[ti] OR "Lobular Carcinoma"[ti] OR "Lobular Carcinomas"[ti] OR "Cancer of Breast"[ti] OR "Cancer of the Breast"[ti] OR "mammary cancer"[ti] OR "mammary cancers"[ti] OR "mammary carcinoma"[ti] OR "mammary carcinomas"[ti] OR "mammary adenocarcinoma"[ti] OR "mammary adenocarcinomas"[ti] OR "mammary tumor"[ti] OR "mammary tumors"[ti] OR "mammary tumour"[ti] OR "mammary tumours"[ti] OR "mammary neoplasm"[ti] OR "mammary neoplasms"[ti] OR "mammary malignancy"[ti] OR "mammary malignancies"[ti] OR (("breast"[ti] OR "breasts"[ti]) AND ("cancer"[ti] OR "cancers"[ti] OR "carcinoma"[ti] OR "carcinomas"[ti] OR "adenocarcinoma"[ti] OR "adenocarcinomas"[ti] OR "tumor"[ti] OR "tumors"[ti] OR "tumour"[ti] OR "tumours"[ti] OR "neoplasm"[ti] OR "neoplasms"[ti] OR "malignancy"[ti] OR "malignancies"[ti] OR "Lobular Carcinoma"[ti] OR "Lobular Carcinomas"[ti]))) AND ("Antigens, CD"[majr] OR "CD Antigen"[ti] OR "CD Antigens"[ti] OR "CD-markers"[ti] OR "CD-marker"[ti] OR "CD1"[ti] OR "CD1d"[ti] OR "CD19"[ti] OR "CD20"[ti] OR "CD34"[ti] OR "CD7"[ti] OR "CD4"[ti] OR "CD8"[ti] OR "CD-1"[ti] OR "CD-1d"[ti] OR "CD-19"[ti] OR "CD-20"[ti] OR "CD-34"[ti] OR "CD-7"[ti] OR "CD-4"[ti] OR "CD-8"[ti] OR "cluster of differentiation"[ti] OR "cluster of designation"[ti] OR "classification determinant"[ti] OR "CD"[ti] OR "CD"[ti] OR "foxp3a protein, zebrafish"[Supplementary Concept] OR "Foxp3 protein, rat"[Supplementary Concept] OR "Foxp3 protein, mouse"[Supplementary Concept] OR "FOXP3 protein, human"[Supplementary Concept] OR "FOXP3"[ti] OR FOXP3*[ti] OR "FOX-P3"[ti] OR FOX-P3*[ti] OR "Forkhead Box P3"[ti] OR "forkhead box protein 3"[ti] OR "forkhead box protein 3a"[ti] OR "Killer Cells, Natural"[Majr] OR "Natural Killer cell"[ti] OR "natural killer cells"[ti] OR "NK cell"[ti] OR "NK cells"[ti] OR "Lymphokine-Activated Killer Cell"[ti] OR "Lymphokine-Activated Killer Cells"[ti] OR "Lymphokine Activated Killer Cells"[ti] OR "LAK Cells"[ti] OR "LAK Cell"[ti]) AND ("Prognosis"[Mesh] OR "Prognosis"[tw] OR prognos*[tw] OR prognost*[tw] OR "Disease-Free Survival"[tw] OR "Progression-Free Survival"[tw] OR "Survival Analysis"[Mesh] OR "overall survival"[tw] OR "Early Diagnosis"[mesh] OR "Early Detection of Cancer"[mesh] OR "early detection"[tw] OR early detect*[tw] OR predict*[tw])) |
| --- | --- |
| Search query tumor-stroma ratio | (("Breast Neoplasms"[Mesh] OR "breast cancer"[tw] OR "breast cancers"[tw] OR "breast carcinoma"[tw] OR "breast carcinomas"[tw] OR "breast adenocarcinoma"[tw] OR "breast adenocarcinomas"[tw] OR "breast tumor"[tw] OR "breast tumors"[tw] OR "breast tumour"[tw] OR "breast tumours"[tw] OR "breast neoplasm"[tw] OR "breast neoplasms"[tw] OR "breast malignancy"[tw] OR "breast malignancies"[tw] OR "Lobular Carcinoma"[tw] OR "Lobular Carcinomas"[tw] OR "Cancer of Breast"[tw] OR "Cancer of the Breast"[tw] OR "mammary cancer"[tw] OR "mammary cancers"[tw] OR "mammary carcinoma"[tw] OR "mammary carcinomas"[tw] OR "mammary adenocarcinoma"[tw] OR "mammary adenocarcinomas"[tw] OR "mammary tumor"[tw] OR "mammary tumors"[tw] OR "mammary tumour"[tw] OR "mammary tumours"[tw] OR "mammary neoplasm"[tw] OR "mammary neoplasms"[tw] OR "mammary malignancy"[tw] OR "mammary malignancies"[tw] OR "breast"[tw] OR "breasts"[tw]) AND ("tumor stroma ratio"[tw] OR "tumor stromal ratio"[tw] OR "tumour stroma ratio"[tw] OR "tumour stromal ratio"[tw] OR "tumor stroma percentage"[tw] OR "tumor stromal percentage"[tw] OR "tumour stroma percentage"[tw] OR "tumour stromal percentage"[tw] OR "carcinoma stroma ratio"[tw] OR "carcinoma stromal ratio"[tw] OR "carcinoma stroma percentage"[tw] OR "carcinoma stromal percentage"[tw] OR "stroma ratio"[tw] OR "stromal ratio"[tw] OR "stroma percentage"[tw] OR "stromal percentage"[tw] OR "stroma cell ratio"[tw] OR "stromal cell ratio"[tw] OR ("Stromal Cells"[Mesh] AND ("ratio"[ti] OR "ratios"[ti])))) |

Supplementary Table 2: Included CD-markers, based on most frequent mentioned markers in the search query

| **Name of marker** | **Type of marker** | **Function of marker** |
| --- | --- | --- |
| CD3 | Protein complex | Is associated with T-cell receptors and involved in recognition of antigens and signal transduction. The complex of CD3 and T-cell receptor is important in the T-cell mediated immune response [1]. |
| CD4 | Glycoprotein | Is found on the surface of lymphocytes. CD4+ T-cells are also involved in the immune response and are thought to play an important role in anti-tumor response [2-4]. |
| CD8 | Glycoprotein on surfaces of lymphocytes | Involved in interactions between cells in the immune system. CD8+ T-cells are involved in the immune response against pathogens, but also against cancer [5-7]. |
| CD24 | Protein | Is expressed on hematopoietic cells, such as B-cells and T-cells. Overexpression of CD24 is seen in several cancer cells, including breast cancer, and could play an important role in the diagnosis and prognosis for cancer [8]. |
| CD44 | Glycoprotein | Marker of cancer stem cells, in both normal and malignant (breast) tissues. The expression of this marker has been studied to monitor progression and prognosis of (breast) cancer [9, 10]. |
| CD68 and CD163 | Glycoprotein | CD68 is especially found in macrophages [11]. CD68 is an example of a tumor associated macrophage marker, along with CD163, which is a macrophage-specific protein [12] [13]. |
| PD-L1 | Protein, ligand | An immune checkpoint inhibitor, which plays a role in the immune response. Higher expression of PD-L1 results in tumor evasion [14] [15]. |
| FOXP3 | Protein | Functions as a regulator in the development and functioning of the immune system and is important in the generation of regulatory T cells (Tregs) [16]. |

Supplementary Table 3: Included articles mentioning CD8 expression and survival in TNBC patients

| Study | Type of breast cancer | Number of patients | Location of expression | Kaplan-Meier analysis | Univariable analysis | Multivariable analysis |
| --- | --- | --- | --- | --- | --- | --- |
| Matsumoto et al. [17] | TNBC | 164 TNBC | Intra-tumoral and stromal | High iCD8 expression was associated with better DFS (p = 0.004) and with better OS (p = 0.02), compared to low CD8 iTILs.  High expression of sCD8 also showed a trend of better DFS, compared to low CD8 sTILs, but these results showed no significant difference (p = 0.136).  This was also the case for OS, where high sCD8 expression showed better survival, compared to low sCD8 expression (p = 0.065). | No additional information | Patients with high CD8 iTILs expression had a better DFS, compared to low CD8 expression (HR 0.48, 95% CI 0.27-0.83, p = 0.0095).  This was also the case for OS, when high iCD8 expression was compared to low iCD8 expression (HR 0.59, 95% CI 0.32-1.07, p = 0.0832).  High sCD8 expression also showed a similar trend of better survival rates, compared to low sCD8 expression, but these results were not statistically significant for both DFS (HR 0.70, 95% CI 0.40-1.21, p = 0.1992) and OS (HR 0.86, 95% CI 0.47-1.57, p = 0.6293). |
| Miyashita et al. [18] | TNBC, treatment with neoadjuvant chemotherapy | 131 | Stromal | According to Kaplan-Meier analysis, patients with high CD8 expression for residual tumor had a better RFS (p < 0.0001) and BCSS (p < 0.0001), compared to the group with low CD8 expression. | Univariable analyses showed that high CD8 expression had a favorable prognostic association with RFS (HR 3.92, 95 % CI 2.016-8.105, p < 0.0001), and BCSS (HR 4.75, 95% CI 2.106-12.12, p = 0.0001). | A high level of CD8 expression was significantly associated with better RFS (HR 3.09, 95 % CI 1.537- 6.614, p = 0.0013), and with better BCSS (HR 3.59, 95% CI 1.499-9.581, p = 0.0036), compared to low CD8 expression. |
| Vihervuori et al. [19] | TNBC | 179 | Entire tumor | Kaplan-Meier curve showed a better survival in patients with high expression of CD8 cells, compared to low expression (p = 0.02, breast cancer specific mortality). | High CD8 expression was significantly correlated with better prognosis (HR 2.1, 95% CI 1.1-4.5, p = 0.02). | In multivariable analysis, high CD8 expression was also a favorable prognostic factor for survival, compared to low expression (HR 1.8, 95% CI 1.1-4.4, p = 0.005). |
| Wang K. et al. [20] | TNBC, HER2 | 42 TNBC, 10 HER2 | Entire tumor and stromal | Not shown | There was no significant association between CD8 expression in the entire tumor or within stroma, and survival outcomes (no p-value mentioned). | No additional information |
| Dieci et al. [21] | TNBC | 244 | Stromal | Kaplan-Meier curve showed better 5 years DFS in the group with high CD8 expression, compared to low CD8 expression (p = 0.001). | High CD8 expression was correlated with better 5-year DFS, compared to the low expression group (HR 0.27, 95% CI 0.13-0.60, p = 0.001). | In multivariable analysis, high CD8 expression was an independent favorable prognostic factor with regard to DFS, compared to low CD8 expression (HR 0.23, 95% CI 0.09-0.59, p = 0.002). |
| Tavares et al. [22] | Non-metastatic TNBC | 76 | Stromal | Kaplan-Meier curve showed that the group with high CD8 expression had a better OS, compared to low CD8 expression. This difference was not statistically significant (p = 0.054). | No additional information | No additional information |
| Wang J. et al. [23] | TNBC | 244 | Stromal | Not shown | There was no significant difference between high and low CD8 expression, regarding OS (HR 0.81, 95% CI 0.50-1.31, p = 0.386), and BCSS (HR 1.08, 95% CI 0.61-1.92, p = 0.785). | No additional information |
| Jafarian et al. [24] | TNBC | 85 | Entire tumor | Not shown | High CD8 expression was associated with better overall survival (p < 0.001). | No additional information |
| Jamiyan et al. [25] | TNBC | 107 | Intra-tumoral and stromal | There was a better OS in patients with high intra-tumoral CD8 expression, compared to low expression, but this was not statistically significant (p = 0.058). Equal trend was seen for RFS (p = 0.057).  In stroma, CD8 expression, high expression was significantly associated with better OS (p = 0.019) and RFS (p = 0.010). | High iCD8 expression seemed correlated with better survival, but the results were not significant for OS (HR 0.304, 95% CI 0.082-1.124, p = 0.074) and RFS (HR 0.303, 95% CI 0.0082-1.119, p = 0.073).  High sCD8 expression was significantly correlated with better OS (HR 0.239, 95% CI 0.065-0.885, p = 0.032) and RFS (HR 0.213, 95% CI 0.058-0.785, p = 0.020). | Compared to low sCD8 expression, high sCD8 expression was an independent prognostic factor for better RFS (HR 0.225, 95% CI 0.061-0.836, p = 0.026) and better OS (HR 0.263, 95% CI 0.071-0.975, p = 0.046) in multivariable analysis. |
| McIntire et al. [26] | TNBC | 74, 2 patients with bilateral TNBC | Entire tumor and stromal | Kaplan-Meier curve of the entire tumor showed a better DFS in patients with high CD8 expression, compared to low, although this difference was not of statistical significance (p > 0.05).  Not shown for stromal expression. | In univariable analysis for the entire tumor, there seemed to be a correlation between CD8 expression and better DFS, but this result was not significant (HR 2.63, 95% CI 0.92-7.48, p = 0.07).  For stromal CD8, there was a significant association between CD8 expression and better DFS (HR 0.27, 95% CI 0.10-0.71, p = 0.008). The results for OS were not significant (HR 0.50, 95% CI 0.24-1.08, p = 0.08). | No additional information |
| Mella et al. [27] | TNBC, ER+, PR+, HER2-) | 95 TNBC | Entire tumor | Kaplan-Meier analysis of TNBC patients showed a better BCSS in the group with high CD8 expression, compared to low expression (p = 0.053). | In TNBC patients, a high CD8 expression was correlated with better BCSS (RR 2.599, 95% CI 1.024-6.596, p = 0.045) | No additional information |
| Aldrees et al. [28] | TNBC | 35 TNBC | Stromal and intra-tumoral | Not shown | Intra-tumoral CD8 expression was not significantly correlated with DFS (HR 0.82, 95% CI 0.28-2.43, p = 0.72) or OS (HR 1.22, 95% CI 0.37-3.96, p = 0.7467).  Stromal CD8 expression also showed no significant correlation with DFS (HR 0.64, 95% CI 0.21-1.93, p = 0.4168) or OS (HR 0.95, 95% CI 0.29-3.13, p = 0.9328). | No additional information |
| Gonzalez-Farre et al. [29] | TNBC | 62 | Entire tumor and intra-tumoral | High CD8 expression in the entire tumor was correlated with better PFS, compared to low expression (HR 6.73, 95% CI 1.30-34.8, p = 0.0086).  High intra-tumoral CD8 expression was associated with better PFS (HR 6.05, 95% CI 1.17-31.3, p = 0.014). | No additional information | No additional information |
| Lopez et al. [30] | Invasive breast cancer, | 144, 56 TNBC | Intra-tumoral | CD8 expression was not related to CSS (p = 0.870). | Intra-tumoral CD8 was not significantly associated with CSS (HR 1.00, 95% CI 0.88-1.14, p = 0.996). | No additional information |
| Salisbury et al. [31] | TNBC | 72 | Intra-tumoral | Not shown | There was no significant difference with regard to PFS and CD8 expression (data not shown). | No additional information |
| Thian et al. [32] | TNBC | 50 | Intra-tumoral | Not shown | CD8 expression was not significantly correlated with OS (p = 0.576). | No additional information |
| Yazaki et al. [33] | Early stage TNBC | 125 | Stromal | Patients with high CD8 expression had a better iDFS, compared to low CD8 expression (p = 0.15). | High CD8 expression was not significantly correlated with iDFS (HR 0.83, 95% CI 0.63-1.08, p = 0.16). | High CD8 expression was an independent prognostic factor for favorable iDFS, compared to low expression (HR 0.74, 95% CI 0.56-0.97, p = 0.03). |

Supplementary Table 4: Included articles mentioning PD-L1 expression and survival in TNBC patients

| Study | Type of breast cancer | Number of patients | Location of expression | Kaplan-Meier analysis | Univariable analysis | Multivariable analysis |
| --- | --- | --- | --- | --- | --- | --- |
| Li Z. et al. [34] | Invasive breast carcinoma | 501: 91  Basal-like | Intra-tumoral | Not shown | In the basal-like subtype, positive PD-L1 expression was associated with worse OS, compared to negative expression (HR 2.835, 95% CI 1.124-7.153, p = 0.027). Similar results were seen for RFS (HR 2.537, 95% CI 1.058-6.082, p = 0.037). | In basal-like subtype, positive PD-L1 expression was an independent prognostic factor for unfavorable OS (HR 2.600, 95% CI 1.016-6.652, p = 0.046).  The results for RFS were similar, although not of statistical significance (HR 2.317, 95% CI 0.956-5.615, p = 0.063). |
| Zhang et al. [35] | TNBC | 58 | Intra-tumoral and stromal | Kaplan-Meier curve showed that the group with high PD-L1 expression intra-tumoral, had a better OS, compared to the group with low expression (p = 0.04).  There was no statistically difference between the groups of high stromal PD-L1 and low PD-L1 expression (p = 0.4). | No additional information | No additional information |
| Dieci et al. [21] | TNBC | 244 | Stromal | The curve of patients with PD-L1 high expression showed a better 5 years DFS, compared to PD-L1 low expression (p = 0.006). | High PD-L1 expression was significantly associated with better 5 years DFS, compared to low PD-L1 expression (HR 0.37, 95% CI 0.18-0.77, p = 0.006). | High PD-L1 expression was an independent prognostic factor for better DFS, compared to low PD-L1 expression (HR 0.28, 95% CI 0.13-0.63, p = 0.002). |
| Tavares et al. [22] | Non-metastatic TNBC | 76 | Intra-tumoral and stromal | In the Kaplan-Meier curve a worse survival was seen for high intra-tumoral PD-L1 expression, but this was not of statistical significance (p = 0.22).  Patients with high PD-L1 expression in the stroma had a higher OS, compared to low PD-L1 expression. However, this difference was also not statistically significant (p = 0.12). | No additional information | No additional information |
| Wang J. et al. [23] | TNBC | 244 | Stromal | Kaplan-Meier curve showed that high stromal PD-L1 expression was correlated with better OS, compared to low expression (HR 0.60, p = 0.034).  The survival curve showed a similar trend of better BCSS in the group with high PD-L1 expression, compared to low, even though this difference was not statistically significant (HR 0.6, p = 0.093). | In the entire cohort, high PD-L1 expression was significantly correlated with better OS compared to low expression (HR 0.60, 95% CI 0.36-0.96, p = 0.034).  With regard to BCSS, there was no significant difference between high and low PD-L1 expression. However, univariate analysis showed a trend of better survival in patients with high PD-L1 expression, compared to low (HR 0.60, 95% CI 0.33-1.09, p = 0.093). | Multivariable analysis showed that high PD-L1 expression was an independent factor for improved OS, compared to low expression (HR 0.54, 95% CI 0.33-0.90, p = 0.018).  With regard to BCSS, high PD-L1 expression was also an independent prognostic factor for better BCSS, compared to low PD-L1 expression (HR 0.50, 95% CI 0.27-0.92, p = 0.026). |
| Zheng J. et al. [36] | TNBC | 159 | Entire tumor | Not shown | Although there was no statistically significant difference, outcomes showed that patients who died had a higher median PD-L1 level, compared to patients who survived. This was the case after 3 years (p = 0.903) and after 5 years (p = 0.383).  There was also no significant difference in overall survival outcomes, between patients with low, medium and high PD-L1 expression (p = 0.903).  At 5 years, there was still no significant difference (p = 0.213). | Multivariate analysis showed that high PD-L1 levels were not an independent prognostic factor with regard to OS, compared to low PD-L1 expression (OR 1.694, 95% CI 0.409-7.026, p = 0.467). |
| Adams et al. [37] | TNBC | 180 | Intra-tumoral | Kaplan-Meier showed a worse OS in the group with high PD-L1 expression, compared to low expression of PD-L1 (p < 0.000) | Univariate analysis showed that high PD-L1 expression was associated with poorer OS, compared to low expression (HR 10.4, 95% CI 3.6-29.6, p = 0.000) | No additional information |
| Arias-Pulido et al. [38] | Inflammatory breast cancer | 221; 44 TNBC | Stromal | In the survival curve of TNBC patients, high stromal PD-L1 expression showed better DFS (p = 0.090) and significantly better BCSS (p = 0.006), compared to low expression. | No additional information | No additional information |
| Chen S. et al. [39] | Local advanced breast cancer after neoadjuvant chemotherapy | 83 TNBC | Intra-tumoral | According to the Kaplan-Meier curve, high PD-L1 expression was associated with worse RFS, compared to low expression (p = 0.002). This was also the case regarding OS (p = 0.006) | Univariable analysis showed a significant correlation between high PD-L1 expression and worse OS (p = 0.006) and worse RFS (p = 0.002). | High PD-L1 expression was an independent unfavorable prognostic factor for both RFS (HR 1.824, 95% CI 1.137-2.926, p = 0.013) and OS (HR 2.585, 95% CI 1.492-4.476, p = 0.001). |
| Dieci et al. [40] | TNBC and HER2-positive metastatic breast cancer | 94; 43 TNBC | Stromal | Kaplan-Meier curve of TNBC patients showed no significant difference in OS between low and high PD-L1 expression (p = 0.936). | No additional information | No additional information |
| Okabe et al. [41] | Luminal types, HER2-positive, TNBC | 21 TNBC | Entire tumor | Kaplan-Meier curve in TNBC patients showed a better OS in patients with positive PD-L1 expression, compared to the negative group. However, this difference was not statistically significant (p = 0.4094). | No additional information | No additional information |
| Yeong J. et al. [42] | TNBC | 269 | Intra-tumoral | Kaplan-Meier curve showed a marginally better OS in patients with high PD-L1 tumor cell expression, compared to low expression (p = 0.070).  With regard to DFS, high PD-L1 tumor cell expression was significantly correlated with better survival outcomes (p = 0.006). | No additional information mentioned | Multivariable analysis showed that high PD-L1 expression was an independent prognostic factor for both better OS (HR 0.40, 95% CI 0.18-0.86, p = 0.020) and better DFS (HR 0.39, 95% CI 0.20-0.76, p = 0.006). |
| Cabioglu et al. [43] | Locally advanced TNBC receiving NAC | 53 | Intra-tumoral, stromal and entire tumor | Not shown | Intra-tumoral PD-L1 expression was not correlated with DFS (p = 0.687) or DSS (0.878).  This was also the case for stromal PD-L1 expression for DFS (p = 0.405) and DSS (p = 0.255).  For the entire tumor, there was again no significant association between PD-L1 expression and DFS (p = 0.822) and DFSS (0.858). | No additional information |
| Salisbury et al. [31] | TNBC | 72 | Entire tumor | Not shown | Based on PD-L1 expression, there was no significant difference with regard to PFS (data not shown). | No additional information |
| Shinohara et al. [44] | Invasive ductal carcinoma | 81, 20 TNBC | Stromal | Patients with high PD-L1 expression in stroma, had a better RFS, compared to low expression, but this was not statistically significant (p = 0.055).  No curve of stromal PD-L1 expression was provided. | No additional information | No additional information |
| Tashireva et al. [45] | TNBC | 30 | Stromal | Not shown | PD-L1 expression was not significantly associated with OS (OR 1.69, 95% CI 0.79-4.28, p = 0.364). |  |
| Yazaki et al. [33] | Early stage TNBC | 125 | Stromal | There was a trend of worse iDFS in patients with positive PD-L1 expression compared to negative expression, but this difference was not statistically significant (p = 0.20). | Positive PD-L1 expression was not significantly associated with iDFS (HR 1.65, 95% CI 0.76-3.5, p = 0.20). | Positive PD-L1 expression was not an independent prognostic factor related to iDFS (HR 1.23, 95% CI 0.52-2.89, p = 0.64). |
| Zhu et al. [46] | TNBC | 176 | Intra-tumoral | Not shown | PD-L1 expression was correlated with worse survival outcomes (no p-values mentioned). |  |

Supplementary Table 5: Included articles mentioning FOXP3 expression and survival in TNBC patients

| Study | Type of breast cancer | Number of patients | Location of expression | Kaplan-Meier analysis/curve | Univariable analysis | Multivariable analysis |
| --- | --- | --- | --- | --- | --- | --- |
| Allaoui et al. [47] | Primary breast cancer types | 498 total  TNBC patients:  315 for BCSS, 313 for RFS | Intra-tumoral | Kaplan-Meier curve showed that TNBC patients with high FOXP3 expression had a worse BCSS, compared to low FOXP3 expression, although this was not statistically significant (p = 0.687). The curve for RFS showed no significant difference between low and high FOXP3 expression (p = 0.739). | No additional information | No additional information |
| Li Z. et al. [34] | Invasive breast carcinoma | 501: 91  Basal-like | Intra-tumoral | Not shown | In TNBC, high FOXP3 expression was correlated with worse OS (HR 7.740, 95% CI 1.044-57.355, p = 0.045) and worse RFS (HR 8.387, 95% CI 1.134-62.036, p = 0.037). | According to multivariate analysis, high FOXP3 expression was an independent prognostic factor for poor OS (HR 8.139, 95% CI 1.070-61.895, p = 0.043).  The results for RFS were similar, but were not statistically significant (HR 6.482, 95% CI 0.838-50.142, p = 0.073). |
| Miyashita et al. [18] | TNBC, treatment with neoadjuvant chemotherapy | 131 | Stromal | Even though the results were not statistically significant, the Kaplan-Meier curves showed a trend of better survival in the group with high FOXP3 expression, compared to the low FOXP3 group with regard to both RFS (p = 0.417) and BCSS (p = 0.175). | According to univariate analysis, there was no significant correlation between FOXP3 expression and RFS. However, the results showed a trend of better RFS in patients with high FOXP3 expression, compared to low (HR 1.30, 95% CI 0.689-2.456, p = 0.4199). this was also the case for BCSS (HR 1.68, 95% CI 0.795-3.699, p = 0.1751). | No additional information |
| Zhang et al. [35] | TNBC | 58 | Entire tumor | Kaplan-Meier curve showed a better OS in the group with high FOXP3 expression, compared to low FOXP3 expression, although it did not reach statistical significance (p = 0.1). | No additional information | No additional information |
| Dieci et al. [21] | TNBC | 244 | Stromal | Kaplan-Meier curve showed better 5 years DFS in the group with high FOXP3 expression, compared to the group with low expression (p = 0.004). | High FOXP3 expression was correlated with more favorable 5 years DFS, compared to low expression (HR 0.48, 95% CI 0.28-0.80, p = 0.004). | High FOXP3 expression was independently correlated with better DFS, compared to low FOXP3 expression (HR 0.47, 95% CI 0.27-0.82, p = 0.007). |
| Koletsa et al. [48] | Intermediate/high-risk operable breast cancer with adjuvant chemotherapy | 122 TNBC | Stromal | Kaplan-Meier curve showed a better DFS in TNBC patients with high sFOXP3 expression, compared to low expression (p = 0.017). The curve for OS in TNBC patients showed similar, better survival in the group with high sFOXP3 expression (p = 0.015). | A high sFOXP3 expression was associated with better DFS in TNBC patients, compared to low expression (HR 0.38, 95% CI 0.17-0.87, p = 0.021).  This was also the case regarding OS in TNBC patients with high sFOXP3 expression (HR 0.35, 95% CI 0.14-0.84, p = 0.021). | Not mentioned |
| Tavares et al. [22] | Non-metastatic TNBC | 76 | Stromal | Kaplan-Meier curve showed that patients with high FOXP3 expression had a better OS, compared to low FOXP3 expression (p = 0.039). | No additional information | No additional information |
| Wang J. et al. [23] | TNBC | 244 | Stromal | Not shown | High FOXP3 expression was not significantly associated, but showed a marginal trend of better OS, compared to low expression (HR 0.68, 95% CI 0.39-1.20, p = 0.182).  There was no association between high and low FOXP3 expression, with regard to BCSS (HR 1.09, 95% CI 0.55-2.19, p = 0.800). | No additional information |
| Adams et al. [37] | TNBC | 180 | Stromal | Kaplan-Meier curve showed significantly worse OS in patients with high FOXP3 expression, compared to high expression (p < 0.000) | High FOXP3 expression was correlated with worse OS (HR 12.7, 95% CI 4.5-35.6, p = 0.000) | No additional information |
| Jamiyan et al. [25] | TNBC | 107 | Intra-tumoral and stromal | The survival curve showed a better RFS in the group with high iFOXP3 expression (p = 0.082). There was also a better OS in the group with high iFOXP3 expression (p = 0.060).  In the group with high sFOXP3 expression, a worse RFS (p = 0.010) and worse OS (p = 0.009) was seen, in comparison with low sFOXP3 expression. | A high iFOXP3 expression was not significantly correlated, but showed a trend of better RFS (HR 0.333, 95% CI 0.090-1.230, p = 0.099), compared to low expression.  This was also the case for OS (HR 0.305, 95% CI 0.082-1.133, p = 0.076).  In contrast, a high sFOXP3 expression was significantly correlated with worse RFS, compared to low expression (HR 5.804, 95% CI 1.265-26.62, p = 0.024). High sFOXP3 was also correlated with worse OS (HR 5.944, 95% CI 1.298-27.22, p = 0.022). | Multivariable analysis showed that high sFOXP3 expression was an independent prognostic factor for worse RFS (HR 7.426, 95% CI 1.596-34.552, p = 0.011) and worse OS (HR 5.467, 95% CI 1.192-25.07, p = 0.029), compared to low FOXP3 expression. |
| Schmidt et al. [49] | High-risk node-negative breast cancer of all types (FinHer trial) and TNBC (HE10/97 trial) | 134 TNBC (FinHer) | Intra-tumoral | Not shown | Univariable analysis showed no significant correlation between FOXP3 expression and DDFS in TNBC patients (HR 1.37, 95% CI 0.63-2.96, p = 0.426). | FOXP3 expression was not an independent prognostic factor for DDFS in TNBC patients according to multivariable analysis (HR 1.54, 95% CI 0.68-3.47, p = 0.300). |
| Aldrees et al. [28] | TNBC | 35 TNBC | Stromal and intra-tumoral | Not shown | Intra-tumoral FOXP3 expression showed no significant correlation with DFS (HR 0.89, 95% CI 0.29-2.67, p = 0.8353), nor with OS (HR 0.657, 95% CI 0.20-2.16, p = 0.4841).  Also no significant association was seen with regard to stromal FOXP3 and DFS (HR 1.65, 95% CI 0.56-4.89, p = 0.3741) and OS (1.88, 95% CI 0.57-6.12, p = 0.3066). | No additional information |
| Chan et al. [50] | Primary invasive breast cancer | 265, 44 TNBC | Intra-tumoral and stromal | In patients with high intra-tumoral FOXP3 expression there was a trend of better survival, but no significant difference for DFS (p = 0.059) and BCSS (p = 0.244).  High stromal FOXP3 expression showed a significant better DFS (p = 0.007) and BCS (p = 0.040) in TNBC patients. | No additional information | No additional information |
| Li, J. et al. [51] | Invasive breast cancer, data from TCGA and PrognoScan database | 123, 25 TNBC | Intra-tumoral | Patients with a high FOXP3 expression had a significant better OS, compared to those with low expression (p = 0.015). | No additional information | No additional information |
| Lopez et al. [30] | Invasive breast cancer | 144, 56 TNBC | Intra-tumoral | Although not statistically significant, there was a trend of better CSS in patients with high FOXP3 expression, compared to low expression (p = 0.139). | FOXP3 expression was not significantly associated with CSS (HR 0.08, 95% CI 0.00-2.86, p = 0.166). | No additional information |

Supplementary Table 6: Included articles mentioning CD3 expression and survival in TNBC patients

| Study | Type of breast cancer | Number of patients | Location of expression | Kaplan-Meier analysis | Univariable analysis | Multivariable analysis |
| --- | --- | --- | --- | --- | --- | --- |
| Allaoui et al. [47] | Primary breast cancer types | TNBC patients:  315 for BCSS, 313 for RFS | Intra-tumoral | Kaplan-Meier curve showed no difference in BCSS between CD3 low and high expression in TNBC patients (p = 0.752). This was the same regarding RFS (p = 0.629) | Not mentioned | Not mentioned |
| Wang J. et al. [23] | TNBC | 244 | Stromal | Not shown | Survival analysis between high and low CD3 expression showed no statistical significant difference for OS (HR 0.84, 95% CI 0.53-1.35, p = 0.479).  The results regarding BCSS also showed no significant difference between high and low CD3 expression (HR 0.96, 95% CI 0.54-1.71, p = 0.881). | Not mentioned |
| Yeong J. et al. [52] | TNBC | 164 | Intra-tumoral and stromal | Kaplan-Meier curve showed a better DFS (p = 0.019) and OS (p = 0.005) in the group with high intra-tumoral CD3 expression, compared to low expression.  A similar correlation was seen in patients with high stromal CD3 expression, compared to low stromal expression, for DFS (p = 0.019) and OS (p = 0.018). | No additional information | Not mentioned |
| Gonzalez-Farre et al. [29] | TNBC | 62 | Entire tumor and intra-tumoral | High CD3 expression in the entire tumor was associated with better PFS (HR 13.5, 95% CI 1.57-116, p = 0.002).  High intra-tumoral CD3 expression also had a significant better PFS, compared to low expression (HR 6.21, 95% CI 1.14-34, p = 0.016). | No additional information | Not mentioned |

Supplementary Table 7: Included articles mentioning CD4 expression and survival in TNBC patients

| Study | Type of breast cancer | Number of patients | Location of expression | Kaplan-Meier analysis | Univariable analysis | Multivariable analysis |
| --- | --- | --- | --- | --- | --- | --- |
| Matsumoto et al. [17] | TNBC | 164 | Intra-tumoral and stromal | There was a significant correlation between high iCD4+ TILs expression and better DFS (p = 0.025), compared to low expression. This was also the case between high versus low iCD4 expression, with regard to OS (p = 0.023).  Furthermore, high sCD4 TILs was significantly associated with better survival for both DFS (p = 0.01), and OS (p = 0.002), compared to low sCD4 expression. | No additional information | High iCD4 expression showed similar favorable survival results, compared to low iCD4 expression, with regard to DFS (HR 0.62, 95% CI 0.36-1.07, p = 0.0843) and OS (HR 0.55, 95% CI 0.30-1.01, p = 0.054).  However, these results were not statistically significant in multivariate Cox regression analysis.  High expression of CD4 sTILs was significantly associated with better DFS (HR 0.46, 95% CI 0.26-0.82, p = 0.0084), and better OS (HR 0.44, 95% CI 0.24-0.83, p = 0.0118), compared to low stromal CD4 expression. |
| Wang K. et al. [20] | TNBC, HER2 | 42 TNBC, 10 HER2 | Entire tumor and stromal | Not shown | There was no significant correlation between CD4 expression in the entire tumor or within the stroma, and survival outcomes. | No additional information |
| Tavares et al. [22] | Non-metastatic TNBC | 76 | Stromal | The group with high CD4 expression showed a better OS in the Kaplan-Meier curve, compared to low CD4 expression, even though this difference was not significant (p = 0.082). | No additional information | No additional information |
| Jamiyan et al. [25] | TNBC | 107 | Intra-tumoral and stromal | Kaplan-Meier curve showed a significant better RFS in patients with high iCD4 expression, in comparison to the low group (p = 0.026). This was also the case for OS (p = 0.038).  Although there was no significant difference, a trend of worse RFS was seen in patients with high sCD4 expression, compared to low (p = 0.253). This trend was also seen regarding OS (p = 0.244). | A high iCD4 expression was correlated with better RFS, compared to low iCD4 expression (HR 0.210, 95% CI 0.046-0.959, p = 0.044).  The results for OS showed a same, marginal trend (HR 0.231, 95% CI 0.051-1.055, p = 0.059).  Although there was no significant difference between low and high sCD4, there was a trend of worse RFS in the group with high sCD4 (HR 1.992, 95% CI 0.599-6.626, p = 0.261).  This was also the case regarding OS (HR 2.014, 95% CI 0.604-6.712, p = 0.254). | Multivariable analysis showed that high iCD4 expression was an independent prognostic factor for longer RFS, compared to low iCD4 expression (HR 0.172, 95% CI 0.037-0.792, p = 0.024) |
| Schmidt et al. [49] | High-risk node-negative breast cancer of all types (FinHer trial) and TNBC (HE10/97 trial) | 134 TNBC in FinHer | Intra-tumoral | Not shown | In TNBC patients, CD4 expression was not significantly associated with DDFS (HR 0.78, 95% CI 0.30-1.55, p = 0.473). | CD4 expression was not an independent prognostic factor for DDFS in TNBC patients according to multivariable analysis in this study (HR 0.89, 95% CI 0.43-1.83, p = 0.744). |
| Lopez et al. [30] | Invasive breast cancer | 144, 56 TNBC | Intra-tumoral | There was no difference with regard to CSS, related to CD4 expression (p = 0.930). | Intra-tumoral CD4 expression was not significantly correlated with CSS (HR 0.95, 95% CI 0.84-1.07, p = 0.401) | No additional information |
| Salisbury et al. [31] | TNBC | 72 | Intra-tumoral | Not shown | There was no significant difference in PFS between groups (data not shown). | No additional information |
| Thian et al. [32] | TNBC | 50 | Intra-tumoral | Not shown | CD4 expression was not significantly correlated with OS (p = 0.699). | No additional information |

Supplementary Table 8: Included articles mentioning CD4/CD8 expression and survival in TNBC patients

| Study | Type of breast cancer | Number of patients | Location of expression | Kaplan-Meier analysis | Univariable analysis | Multivariable analysis |
| --- | --- | --- | --- | --- | --- | --- |
| Matsumoto et al. [17] | TNBC | 164 | Intra-tumoral and stromal | Expression of high CD4+/CD8+ iTILs was significantly associated with better DFS (p = 0.023) and with better OS (p = 0.027). This was in comparison with the other subgroups (CD4-low/CD8-low, CD4-high/CD8-low, and CD4-low/CD8-high expression).  High expression of CD4+/CD8+ sTILs was also significantly correlated with improved OS (p = 0.021), compared to the other subgroups. | No additional information | The combination of high CD4 and CD8 iTILs expression showed a significantly improved DFS (HR 0.43, 95% CI 0.22-0.83, p = 0.0121), compared to the CD4-low/CD8-low subgroup.  High expression of CD4/CD8 was also correlated with improved OS (HR 0.46, 95% CI 0.23-0.94, p = 0.0329), compared to low CD4/CD8.  A combination of CD4 and CD8 sTILs only showed a significantly better DFS (HR of 0.49 (95% CI 0.26-0.93, p = 0.0295), compared to the low CD4/CD8 group.  Even though the results were not statistically significant, the high CD4/CD8 expression group showed a more favorable OS, compared to the low CD4/CD8 group (HR 0.53, 95% CI 0.26-1.09, p = 0.0824). |
| Wang K. et al. [20] | TNBC, HER2 | 42 TNBC, 10 HER2 | Entire tumor and stromal | Kaplan-Meier curve of expression in the entire tumor showed a better RFS in the group with a high CD4/CD8 ratio, compared to a low ratio (no p-value mentioned in curve).  The curve with regard to OS, showed a similar trend with better survival in the group with high CD4/CD8 ratio (no p-value mentioned in curve). | In the TNBC patients, a high ratio of CD4/CD8 expression in the entire tumor was correlated with better RFS, compared to low expression (HR 0.16, 95% CI 0.04-0.38, p = 0.0004). This was also the case with regard to OS (HR 0.10, 95% CI 0.03-0.32, p = 0.0002).  In the stroma, there was a similar trend of more favorable RFS in the group with a high CD4/CD8 ratio, although the results were not statistically significant (HR 0.70, 95% CI 0.24-2.02, p = 0.52). The results for OS showed a similar outcome (HR 0.73, 95% CI 0.22-2.39, p = 0.60). | No additional information |
| Jafarian et al. [24] | TNBC | 85 | Entire tumor | Not shown | A high CD4/CD8 expression was associated with worse overall survival (p = 0.008). | No additional information |
| Jamiyan et al. [25] | TNBC | 107 | Intra-tumoral and stromal | Kaplan-Meier curve showed no significant difference between low and high iCD4/CD8 expression. However, the curve showed a better RFS in the group with high iCD4/CD8 expression (p = 0.118). A similar trend was seen regarding OS (p = 0.171).  Even though the difference was not statistically significant, the survival curve showed a marginal worse RFS in patients with high sCD4/CD8 expression, compared to low (p = 0.327). The same marginal trend was seen in the curve regarding OS (p = 0.423). | High iCD4/CD8 expression showed a trend of better OS, but this was not of statistical significance (HR 0.413, 95% CI 0.111-1.530, p = 0.186).  This was also the case for RFS (HR 0.369, 95% CI 0.100-1.365, p = 0.135).  High sCD4/CD8 expression showed a correlation with worse OS, compared to low expression (HR 1.368, 95% CI 1.042-1.797, p = 0.024).  This was also seen regarding RFS (HR 1.362, 95% CI 1.049-1.769, p = 0.021). | In multivariable analysis, sCD4/CD8 expression was not an independent prognostic factor for OS (HR 1.059, 95% CI 0.673-1.665, p = 0.804).  This was also the case for RFS (HR 1.003, 95% CI 0.650-1.549, p = 0.988). |
| Thian et al. [32] | TNBC | 50 | Intra-tumoral | High CD4/CD8 ratio (high CD4 expression to low CD8 expression) was associated with better DFS survival, compared to low ratio (p-value not mentioned). | High CD4/CD8 ratio was not significantly correlated with OS (p = 0.055). | No additional information |

Supplementary Table 9: Included articles mentioning CD68 expression and survival in TNBC patients

| Study | Type of breast cancer | Number of patients | Location of expression | Kaplan-Meier analysis | Univariable analysis | Multivariable analysis |
| --- | --- | --- | --- | --- | --- | --- |
| Tavares et al. [22] | Non-metastatic TNBC | 76 | Stromal | Kaplan-Meier curve showed that high CD68 expression had a better OS, compared to low CD68 expression, but this association was not significant (p = 0.074). | No additional information | No additional information |
| Wang J. et al. [23] | TNBC | 244 | Stromal | Not shown | There was no significant difference between high and low CD68 expression, with regard to both OS (HR 0.99, 95% CI 0.61-1.60, p = 0.968), and BCSS (HR 0.82, 95% CI 0.46-1.47, p = 0.507). | No additional information |
| Pelekanou et al. [53] | Cohort A: ER+ and ER-  Cohort B: TNBC | 160 TNBC | Entire tumor | In TNBC patients, there was no difference in OS with regard to CD68 expression (p = 0.21). | No additional information | In cohort B, CD68 expression was not an independent prognostic factor for OS. However, CD68 expression showed a trend of worse survival in TNBC patients (HR 13.44, 95% CI 0.76-382.29, p = 0.07). |
| Jaaskelainen et al. [54] | Primary, early breast cancer, HER2+ and cohort HER2- | 278, 17 TNBC | Intra-tumoral | Not shown | There was no significant correlation found between CD68 expression and survival, with regard to OS (HR 1.17, 95% CI 0.62-2.20, p = 0.621), BCSS (HR 1.75, 95% CI 0.66-4.63, p = 0.262), or DFS (HR 1.75, 95% CI 0.66-4.63, p = 0.262). | Not mentioned |
| Lopez et al. [30] | Invasive breast cancer | 144, 56 TNBC | Intra-tumoral | Patients with high CD68 expression showed a trend of better CSS, compared to low expression (p = 0.139). | No additional information | No additional information |
| Shinohara et al. [44] | Invasive ductal carcinoma | 81, 20 TNBC | Intra-tumoral and stromal | Stromal high CD68 expression was associated with better RFS, compared to low expression, although the difference was not statistically significant (p = 0.055).  Kaplan Meier curve of intra-tumoral expression showed better RFS in patients with high CD68 expression, but again not a significant difference (p = 0.273). | No additional information | No additional information |

Supplementary Table 10: Included articles mentioning CD163 expression and survival in TNBC patients

| Study | Type of breast cancer | Number of patients | Location of expression | Kaplan-Meier analysis | Univariable analysis | Multivariable analysis |
| --- | --- | --- | --- | --- | --- | --- |
| Tavares et al. [22] | Non-metastatic TNBC | 76 | Stromal | High CD163 expression showed a trend of better OS in the Kaplan-Meier curve, but there was no significant correlation (p = 0.13). | No additional information | No additional information |
| Adams et al. [37] | TNBC | 180 | Stromal | Kaplan-Meier curve showed that the group with high CD163 expression had a worse OS, compared to low expression (p = 0.0005) | High CD163 expression was associated with worse OS (HR 3.2, 95% CI 1.7-6.2, p = 0.0005) | No additional information |
| Pelekanou et al. [53] | Cohort A: ER+ and ER-  Cohort B: TNBC | 160 | Entire tumor | Kaplan Meier curve of TNBC patients showed that high CD163 expression was associated with better OS, compared to low expression (p = 0.04). | No additional information | In cohort B (TNBC patients), multivariable analysis showed no significant correlation between CD163 expression and OS (HR 0.45, 95% CI 0.08-2.25, p = 0.33). |
| Jaaskelainen et al. [54] | Primary, early breast cancer, HER2+ and cohort HER2- | 278, 17 TNBC | Intra-tumoral | Not shown | CD163 expression was not associated with OS (HR 1.30, 95% CI 0.69-2.44, p = 0.418), BCSS (HR 1.16, 95% CI 0.51-2.63, p = 0.721) and DFS (HR 1.16, 95% CI 0.51-2.63, p = 0.721). | No additional information |
| Shinohara et al. [44] | Invasive ductal carcinoma | 81, 20 TNBC | Intra-tumoral and stromal | Patients with high stromal CD163 expression had a better RFS, compared to low expression, but this difference was not significant (p =0.397).  High intra-tumoral CD163 expression showed a worse RFS instead, but again this was not statistically significant (p = 0.346). | No additional information | No additional information |

Supplementary Table 11: Included articles mentioning CD44/CD24 expression and survival in TNBC patients

| Study | Type of breast cancer | Number of patients | Location of expression | Kaplan-Meier analysis | Univariable analysis | Multivariable analysis |
| --- | --- | --- | --- | --- | --- | --- |
| Wang H. et al. [55] | TNBC | 145 | Intra-tumoral | Kaplan-Meier curve showed worse OS in patients with CD44+/CD24- expression, compared to patients with CD44-/CD24- expression (p = 0.005). | No additional information | CD44+/CD24- expression was significantly correlated with worse OS, compared to patients with CD44-/CD24- expression (HR 4.38, 95% CI 1.57-12.18, p = 0.005). This was the same for DFS, although these results were not statistically significant (HR 2.38, 95% CI 0.90-6.33, p = 0.081). |
| Qiao et al. [56] | Invasive breast cancer | 221 TNBC | Intra-tumoral | In TNBC patients, high CD44+/CD24- expression was correlated with worse DFS, compared to low CD44+/CD24- expression (p < 0.0001). | Not mentioned for TNBC | No additional information |
| Chekhun et al. [57] | Stage I-II breast cancer | 29 basal like | Intra-tumoral | Survival of patients with basal like breast cancer was worse in the group with positive CD44+/CD24- expression, compared to absence of these cells (p < 0.05). | No additional information | No additional information |

Supplementary Table 12: Included articles mentioning CD24 expression and survival in TNBC patients

| Study | Type of breast cancer | Number of patients | Location of expression | Kaplan-Meier analysis | Univariable analysis | Multivariable analysis |
| --- | --- | --- | --- | --- | --- | --- |
| Kwon et al. [58] | Primary invasive breast cancer | 183 TNBC | Intra-tumoral | Although not statistically significant, patients with high CD24 expression showed a trend of worse OS, compared to low expression in TNBC patients (p = 0.091).  Kaplan-Meier curve showed a worse 5-year DFS in patients with CD24 high expression, compared to low. However, this difference was not statistically significant (p = 0.184). This trend was also seen for 10-year DFS (p = 0.087) and 15-year DFS (p = 0.087).  High CD24 expression in TNBC patients was associated with worse 5-year DMFS, although this difference was not of statistical significance (p = 0.159).  The curve for 10-year and 15-year DMFS showed a marginal trend of worse survival in high CD24 expression (p = 0.058 for both). In the survival curve independent of time, there was a worse DMFS in patients with CD24 high expression, compared to low expression (p = 0.058). | No additional information | According to multivariable analysis, high CD24 expression was an independent prognostic factor for unfavorable DFS (HR 2.05, 95% CI 1.02-4.14, p = 0.045), for worse OS (HR 1.98, 95% CI 1.02-3.85, p = 0.044), and for poor DMFS (HR 2.18, 95% CI 1.07-4.45, p = 0.032). |
| Zhu et al. [46] | TNBC | 176 | Entire tumor | Patients with positive CD24 expression had a significant worse DFS (p < 0.001) and OS (p = 0.010), compared to patients with negative CD24 expression. | CD24 expression was significantly associated with worse DFS (p < 0.001) and OS (p = 0.010). | No additional information |

Supplementary Table 13: Included articles mentioning CD44 expression and survival in TNBC patients

| Study | Type of breast cancer | Number of patients | Location of expression | Kaplan-Meier analysis | Univariable analysis | Multivariable analysis |
| --- | --- | --- | --- | --- | --- | --- |
| Tokunaga et al. [59] | TNBC, with neoadjuvant chemotherapy | 48 | Intra-tumoral | Kaplan-Meier curve showed a poorer DMFS in patients with high CD44v9 expression, compared to low expression (p = 0.0291).  The curve  of CD44v9 expression before neoadjuvant chemotherapy showed that the group with high CD44v9 expression had a worse DMFS, compared to low expression (p = 0.0333).  After NAC, there was no significant difference between both group with regard to DMFS (p = 0.4260). | Univariable analysis showed that a high CD44v9 expression before NAC was associated with worse DMFS (HR 6.86, 95% CI 1.31-126.1, p = 0.0189).  High CD44v9 expression after NAC was not significantly associated with DMFS (HR 0.61, 95% CI 0.18-2.33, p = 0.4427). | In multivariable analysis, a high CD44v9 expression before NAC was not an independent prognostic factor for DMFS (HR 2.23, 95% CI 0.22-55.8, p = 0.5196). |

Supplementary Table 14: Included articles mentioning TSR and survival

| Study | Type of breast cancer | Number of patients | Kaplan Meier analysis | Univariable analysis | Multivariable analysis |
| --- | --- | --- | --- | --- | --- |
| De Kruijf et al. [60] | Non-metastatic invasive breast cancer | 574, 82 TNBC | The curve of TNBC patients showed worse RFP in stroma-rich patients (p = 0.002). There was also a trend of worse OS, but this result showed no statistically significant difference (p = 0.085). | In TNBC patients, a rich stroma tumor was significantly correlated with worse RFP (HR 3.91, 95% CI 1.49-6.83, p = 0.003). The group with stroma-rich tumors showed a trend towards worse OS, compared to stroma-poor, but this result was not statistically significant (HR 1.60, 95% CI 0.932-2.736, p = 0.088). | In TNBC patients, stroma-rich tumors were associated with significantly poorer RFP (HR 2.92, 95% CI 1.358-6.320, p = 0.006) and worse OS (HR 1.87, 95% CI 1.071-3.260, p = 0.028). |
| Moorman et al. [61] | TNBC | 124 | Kaplan Meier curve showed a worse RFP in patients with high stroma, compared to low stroma (p = 0.004). The stroma high group also had a worse OS (p = 0.035). | Stroma-high patients had a significantly worse RFP (HR 2.93; 95% CI 1.37-6.26; p = 0.004) and OS (HR 2.56; 95% CI 1.03-6.35; p = 0.035) compared to stroma-low patients for 5-year follow-up. | High stroma was an independent prognostic factor for poorer RFP (HR 2.39, 95% CI 1.07-5.29, p = 0.033) and OS (HR 3.00, 95% CI 1.08-8.32, p = 0.034). |
| Dekker et al. [62] | Patients with T1– T3, N0-2, and M0 breast cancer who were not previously treated | 403, 69 TNBC | In TNBC patients, high stroma was also associated with worse DFS (p = 0.043). | In TNBC patients, high stroma was correlated with worse DFS (HR 2.21, 95% CI 1.00-4.84, p = 0.049). | In TNBC patients, TSR was an independent prognostic factor for worse DFS (HR 2.71, 95% CI 1.11-6.61, p = 0.028). |
| Gujam et al. [63] | Primary operable invasive ductal breast cancer | 361, 151 TNBC | High stroma percentage correlated with worse cancer-specific survival in TNBC patients (p = 0.035). | In TNBC, high stroma was correlated with worse CSS (HR 1.06, 95% CI 1.03-1.12, p = 0.035). | In TNBC patients, there was no significant difference in multivariable analysis with regard to stromal content (p = 0.151, no HR mentioned). |
| Millar et al. [64] | Luminal ER+ and TNBC | 647: 403 luminal ER, 244 TNBC | Kaplan-Meier curve of TNBC patients showed that high stroma was correlated with worse BCSS, compared to low stroma (p = 0.014). | In univariable analysis, there was a trend of worse OS in the group with high stroma, although this difference was not significant (HR 1.54, 95% CI 0.93-2.55, p = 0.093).  With regard to BCSS, high stroma was significantly associated with worse survival (HR 2.34, 95% CI 1.19-4.59, p = 0.014). | In TNBC patients, multivariable analysis showed that high stroma was significantly associated with worse OS (HR 1.90, 95% CI 1.10-3.29, p = 0.021) and worse BCSS (HR 2.64, 95% CI 1.31-5.35, p = 0.007). |
| Zakhartseva et al. [65] | TNBC | 232 | Kaplan-Meier curve showed that patients with high tumor stroma had a worse OS (p = 0.008) and worse DFS (p = 0.03), compared to patients with low stroma. | Patients with stroma-high tumors had a worse OS, compared to stroma-low tumors (HR 1.89, 95% CI 1.18-3.03, p = 0.008). Similar results were seen with regard to DFS (HR 1.55, 95% CI 1.05-2.28, p = 0.027). | Multivariable analysis showed that high stroma was an independent prognostic factor for worse OS (HR 2.11, 95% CI 1.29-3.43, p = 0.002) and DFS (HR 1.83, 95% CI 1.22-2.78, p = 0.004). |
| Hacking et al. [66] | TNBC, treated with NAC | 120 | Not shown | High stroma was associated with poor clinical outcome (HR 3.79, 95% CI 1.66-8.64, p = 0.002). | Not mentioned |
| Yan et al. [67] | Invasive breast cancer | 240, 63 TNBC | Patients with high stroma had a worse DFS, compared to patients with low stroma (p = 0.003). | High stroma was significantly associated with worse DFS, compared to low stroma (HR 2.59, 95% CI 1.34-5.03, p = 0.005). | High stroma was an independent prognostic factor for worse DFS (HR 2.68, 95% CI 1.29-5.59, p = 0.008). |

**Acknowledgments**

We would like to thank J.W. Schoones from the Walaeus Library of the Leiden University for his help in the conduction of the search query, and the Bollenstreekfonds for financially supporting this research.

**Conflicts of interest**

The authors declare no potential conflicts of interest.

**References**

1. Yang, H., R.M. Parkhouse, and T. Wileman, *Monoclonal antibodies that identify the CD3 molecules expressed specifically at the surface of porcine gammadelta-T cells.* Immunology, 2005. **115**(2): p. 189-96.

2. Wang, R.-F., G.C. Prendergast, and E.M. Jaffee, *Chapter 15 - Regulatory T cells in Tumor Immunity: Role of Toll-Like Receptors*, in *Cancer Immunotherapy*. 2007, Academic Press: Burlington. p. 277-287.

3. Robins, H., et al., *CD4+ and CD8+ T cell β antigen receptors have different and predictable V and J gene usage and CDR3 lengths (115.10).* The Journal of Immunology, 2012. **188**(1 Supplement): p. 115.10-115.10.

4. Tay, R.E., E.K. Richardson, and H.C. Toh, *Revisiting the role of CD4+ T cells in cancer immunotherapy—new insights into old paradigms.* Cancer Gene Therapy, 2021. **28**(1): p. 5-17.

5. Actor, J.K., *Chapter 4 - T Lymphocytes: Ringleaders of Adaptive Immune Function*, in *Introductory Immunology (Second Edition)*. 2019, Academic Press. p. 45-62.

6. Gao, G.F. and B.K. Jakobsen, *Molecular interactions of coreceptor CD8 and MHC class I: the molecular basis for functional coordination with the T-cell receptor*, in *Immunol Today*. 2000: England. p. 630-6.

7. Raskov, H., et al., *Cytotoxic CD8+ T cells in cancer and cancer immunotherapy.*

8. Fang, X., et al., *CD24: from A to Z.* Cellular & Molecular Immunology, 2010. **7**(2): p. 100-103.

9. Li, W., et al., *Unraveling the roles of CD44/CD24 and ALDH1 as cancer stem cell markers in tumorigenesis and metastasis.* Sci Rep, 2017. **7**(1): p. 13856.

10. Jang, M.H., et al., *Clinicopathological analysis of CD44 and CD24 expression in invasive breast cancer.* Oncol Lett, 2016. **12**(4): p. 2728-2733.

11. Chistiakov, D.A., et al., *CD68/macrosialin: not just a histochemical marker.* Laboratory Investigation, 2017. **97**(1): p. 4-13.

12. Ni, C., et al., *CD68- and CD163-positive tumor infiltrating macrophages in non-metastatic breast cancer: a retrospective study and meta-analysis.* J Cancer, 2019. **10**(19): p. 4463-4472.

13. Etzerodt, A. and S.K. Moestrup, *CD163 and inflammation: biological, diagnostic, and therapeutic aspects.* Antioxid Redox Signal, 2013. **18**(17): p. 2352-63.

14. Benicky, J., et al., *PD-L1 Glycosylation and Its Impact on Binding to Clinical Antibodies.* J Proteome Res, 2021. **20**(1): p. 485-497.

15. Han, Y., D. Liu, and L. Li, *PD-1/PD-L1 pathway: current researches in cancer.* Am J Cancer Res, 2020. **10**(3): p. 727-742.

16. Takenaka, M., et al., *FOXP3 expression in tumor cells and tumor-infiltrating lymphocytes is associated with breast cancer prognosis.* Mol Clin Oncol, 2013. **1**(4): p. 625-632.

17. Matsumoto, H., et al., *Increased CD4 and CD8-positive T cell infiltrate signifies good prognosis in a subset of triple-negative breast cancer.* Breast Cancer Res Treat, 2016. **156**(2): p. 237-47.

18. Miyashita, M., et al., *Prognostic significance of tumor-infiltrating CD8+ and FOXP3+ lymphocytes in residual tumors and alterations in these parameters after neoadjuvant chemotherapy in triple-negative breast cancer: a retrospective multicenter study.* Breast Cancer Res, 2015. **17**(1): p. 124.

19. Vihervuori, H., et al., *Tumor-infiltrating lymphocytes and CD8(+) T cells predict survival of triple-negative breast cancer.* J Cancer Res Clin Oncol, 2019. **145**(12): p. 3105-3114.

20. Wang, K., et al., *The CD4/CD8 ratio of tumor-infiltrating lymphocytes at the tumor-host interface has prognostic value in triple-negative breast cancer.* Hum Pathol, 2017. **69**: p. 110-117.

21. Dieci, M.V., et al., *Integration of tumour infiltrating lymphocytes, programmed cell-death ligand-1, CD8 and FOXP3 in prognostic models for triple-negative breast cancer: Analysis of 244 stage I-III patients treated with standard therapy*, in *Eur J Cancer*. 2020, © 2020 The Author(s). Published by Elsevier Ltd.: England. p. 7-15.

22. Tavares, M.C., et al., *A high CD8 to FOXP3 ratio in the tumor stroma and expression of PTEN in tumor cells are associated with improved survival in non-metastatic triple-negative breast carcinoma.* BMC Cancer, 2021. **21**(1): p. 901.

23. Wang, J., et al., *Multiplexed immunofluorescence identifies high stromal CD68+PD-L1+ macrophages as a predictor of improved survival in triple negative breast cancer.* Scientific Reports, 2021. **11**(1): p. 21608.

24. Jafarian, A.H., et al., *Evaluation of CD30/CD4/CD8 in triple-negative invasive ductal carcinoma of breast in association with clinicopathological prognostic factors.* Indian J Pathol Microbiol, 2018. **61**(4): p. 500-504.

25. Jamiyan, T., et al., *Prognostic impact of a tumor-infiltrating lymphocyte subtype in triple negative cancer of the breast.* Breast Cancer., 2020.

26. McIntire, P.J., et al., *Hot Spot and Whole-Tumor Enumeration of CD8(+) Tumor-Infiltrating Lymphocytes Utilizing Digital Image Analysis Is Prognostic in Triple-Negative Breast Cancer.* Clin Breast Cancer, 2018. **18**(6): p. 451-458.e1.

27. Mella, M., et al., *Tumor infiltrating CD8(+) T lymphocyte count is independent of tumor TLR9 status in treatment naïve triple negative breast cancer and renal cell carcinoma.* Oncoimmunology, 2015. **4**(6): p. e1002726.

28. Aldrees, R., G.P. Siegal, and S. Wei, *The Peritumoral CD8+/FOXP3+ Cell Ratio Has Prognostic Value in Triple-negative Breast Cancer.* Applied Immunohistochemistry & Molecular Morphology, 2023. **31**(9): p. 621-628.

29. Gonzàlez-Farré, M., et al., *Characterization and spatial distribution of the immune cell infiltrate in triple-negative breast cancer: a novel classification based on plasma cells and CD8+ T cells.* Human Pathology, 2023. **139**: p. 91-105.

30. López, C., et al., *CD68 and CD83 immune populations in non-metastatic axillary lymph nodes are of prognostic value for the survival and relapse of breast cancer patients.* Breast Cancer, 2022. **29**(4): p. 618-635.

31. Salisbury, T., et al., *Histological subtype is associated with PD-L1 expression and CD8+ T-cell infiltrates in triple-negative breast carcinoma.* Annals of Diagnostic Pathology, 2022. **57**: p. 151901.

32. Thian, Q.Z., et al., *High CD4+/CD8+ intratumour ratio is associated with favourable outcome in triple-negative breast cancer.* Medicine & Health, 2022. **17**(2): p. 101-124.

33. Yazaki, S., et al., *Integrative prognostic analysis of tumor-infiltrating lymphocytes, CD8, CD20, programmed cell death-ligand 1, and tertiary lymphoid structures in patients with early-stage triple-negative breast cancer who did not receive adjuvant chemotherapy.* Breast Cancer Res Treat, 2023. **197**(2): p. 287-297.

34. Li, Z., et al., *PD-L1 expression is associated with tumor FOXP3<sup>+</sup>regulatory T-cell infiltration of breast cancer and poor prognosis of patient.* Journal of Cancer, 2016. **7**(7): p. 784-793.

35. Zhang, L., et al., *The predictive and prognostic value of Foxp3+/CD25+ regulatory T cells and PD-L1 expression in triple negative breast cancer.* Ann Diagn Pathol, 2019. **40**: p. 143-151.

36. Zheng, J., et al., *Higher CD1a Levels Correlate with PD-L1 Expression and Predict Worse Overall Survival in Triple-Negative Breast Carcinoma.* Breast Care (Basel), 2022. **17**(1): p. 31-39.

37. Adams, T.A., et al., *Composite analysis of immunological and metabolic markers defines novel subtypes of triple negative breast cancer.* Modern Pathology, 2018. **31**(2): p. 288-298.

38. Arias-Pulido, H., et al., *The combined presence of CD20 + B cells and PD-L1 + tumor-infiltrating lymphocytes in inflammatory breast cancer is prognostic of improved patient outcome.* Breast Cancer Res Treat, 2018. **171**(2): p. 273-282.

39. Chen, S., et al., *PD-L1 expression of the residual tumor serves as a prognostic marker in local advanced breast cancer after neoadjuvant chemotherapy.* Int J Cancer, 2017. **140**(6): p. 1384-1395.

40. Dieci, M.V., et al., *Immune characterization of breast cancer metastases: prognostic implications.* Breast Cancer Res, 2018. **20**(1): p. 62.

41. Okabe, M., et al., *Predictive factors of the tumor immunological microenvironment for long-term follow-up in early stage breast cancer.* Cancer Science, 2017. **108**(1): p. 81-90.

42. Yeong, J., et al., *Prognostic value of CD8 + PD-1+ immune infiltrates and PDCD1 gene expression in triple negative breast cancer.* J Immunother Cancer, 2019. **7**(1): p. 34.

43. Cabioglu, N., et al., *Diverging prognostic effects of CD155 and CD73 expressions in locally advanced triple-negative breast cancer.* Front Oncol, 2023. **13**: p. 1165257.

44. Shinohara, H., et al., *Spatial and Quantitative Analysis of Tumor-Associated Macrophages: Intratumoral CD163-/PD-L1+ TAMs as a Marker of Favorable Clinical Outcomes in Triple-Negative Breast Cancer.* Int J Mol Sci, 2022. **23**(21).

45. Tashireva, L.A., et al., *B Lymphocytes Are a Predictive Marker of Eribulin Response and Overall Survival in Locally Advanced or Metastatic Breast Cancer: A Multicenter, Two-Cohort, Non-Randomized, Open-Label, Retrospective Study.* Front Oncol, 2022. **12**: p. 909505.

46. Zhu, X., et al., *CD24 May Serve as an Immunotherapy Target in Triple-Negative Breast Cancer by Regulating the Expression of PD-L1.* Breast Cancer (Dove Med Press), 2023. **15**: p. 967-984.

47. Allaoui, R., et al., *Infiltration of gammadelta T cells, IL-17+ T cells and FoxP3+ T cells in human breast cancer.* Cancer Biomark, 2017. **20**(4): p. 395-409.

48. Koletsa, T., et al., *Prognostic impact of stromal and intratumoral CD3, CD8 and FOXP3 in adjuvantly treated breast cancer: do they add information over stromal tumor-infiltrating lymphocyte density?*, in *Cancer Immunol Immunother*. 2020: Germany. p. 1549-1564.

49. Schmidt, M., et al., *Prognostic impact of CD4-positive T cell subsets in early breast cancer: a study based on the FinHer trial patient population.* Breast Cancer Res, 2018. **20**(1): p. 15.

50. Chan, R., et al., *Spatial Distribution and Densities of CD103+ and FoxP3+ Tumor Infiltrating Lymphocytes by Digital Analysis for Outcome Prediction in Breast Cancer.* Oncologist, 2024. **29**(3): p. e299-e308.

51. Li, J., et al., *The expression landscape of FOXP3 and its prognostic value in breast cancer.* Ann Transl Med, 2022. **10**(14): p. 801.

52. Yeong, J., et al., *Higher densities of Foxp3(+) regulatory T cells are associated with better prognosis in triple-negative breast cancer.* Breast Cancer Res Treat, 2017. **163**(1): p. 21-35.

53. Pelekanou, V., et al., *CD68, CD163, and matrix metalloproteinase 9 (MMP-9) co-localization in breast tumor microenvironment predicts survival differently in ER-positive and -negative cancers.* Breast Cancer Res, 2018. **20**(1): p. 154.

54. Jääskeläinen, M.M., et al., *High Numbers of CD163+ Tumor-Associated Macrophages Predict Poor Prognosis in HER2+ Breast Cancer.* Cancers (Basel), 2024. **16**(3).

55. Wang, H., et al., *CD44(+)/CD24(-) phenotype predicts a poor prognosis in triple-negative breast cancer.* Oncol Lett, 2017. **14**(5): p. 5890-5898.

56. Qiao, X., et al., *Association of human breast cancer CD44(-)/CD24(-) cells with delayed distant metastasis.* Elife, 2021. **10**.

57. Chekhun, S.V., et al., *СD44+/CD24- markers of cancer stem cells in patients with breast cancer of different molecular subtypes.* Exp Oncol, 2015. **37**(1): p. 58-63.

58. Kwon, M.J., et al., *CD24 Overexpression Is Associated with Poor Prognosis in Luminal A and Triple-Negative Breast Cancer.* PLoS One, 2015. **10**(10): p. e0139112.

59. Tokunaga, E., et al., *CD44v9 as a poor prognostic factor of triple-negative breast cancer treated with neoadjuvant chemotherapy.* Breast Cancer, 2019. **26**(1): p. 47-57.

60. de Kruijf, E.M., et al., *Tumor-stroma ratio in the primary tumor is a prognostic factor in early breast cancer patients, especially in triple-negative carcinoma patients.* Breast Cancer Res Treat, 2011. **125**(3): p. 687-96.

61. Moorman, A.M., et al., *The prognostic value of tumour-stroma ratio in triple-negative breast cancer.* Eur J Surg Oncol, 2012. **38**(4): p. 307-13.

62. Dekker, T.J., et al., *Prognostic significance of the tumor-stroma ratio: validation study in node-negative premenopausal breast cancer patients from the EORTC perioperative chemotherapy (POP) trial (10854).* Breast Cancer Res Treat, 2013. **139**(2): p. 371-9.

63. Gujam, F.J., et al., *The relationship between the tumour stroma percentage, clinicopathological characteristics and outcome in patients with operable ductal breast cancer.* Br J Cancer, 2014. **111**(1): p. 157-65.

64. Millar, E.K., et al., *Tumour Stroma Ratio Assessment Using Digital Image Analysis Predicts Survival in Triple Negative and Luminal Breast Cancer.* Cancers (Basel), 2020. **12**(12).

65. Zakhartseva, L.M. and M.A. Yanovytska, *PROGNOSTIC VALUE OF TUMOR STROMA RATIO IN TRIPLE NEGATIVE BREAST CANCER.* Wiad Lek, 2021. **74**(3 cz 2): p. 565-571.

66. Hacking, S.M., et al., *Whole slide image features predict pathologic complete response and poor clinical outcomes in triple-negative breast cancer.* Pathology - Research and Practice, 2023. **246**: p. 154476.

67. Yan, D., et al., *Tumour stroma ratio is a potential predictor for 5-year disease-free survival in breast cancer.* BMC Cancer, 2022. **22**(1): p. 1082.
